# Federated Learning in Healthcare: A Benchmark Comparison of Engineering and Statistical Approaches for Structured Data Analysis

**DOI:** 10.34133/hds.0196

**Published:** 2024-12-04

**Authors:** Siqi Li, Di Miao, Qiming Wu, Chuan Hong, Danny D’Agostino, Xin Li, Yilin Ning, Yuqing Shang, Ziwen Wang, Molei Liu, Huazhu Fu, Marcus Eng Hock Ong, Hamed Haddadi, Nan Liu

**Affiliations:** ^1^ Centre for Quantitative Medicine, Duke-NUS Medical School, Singapore, Singapore.; ^2^ Department of Biostatistics and Bioinformatics, Duke University, Durham, NC, USA.; ^3^ Department of Biostatistics, Columbia University Mailman School of Public Health, New York, NY, USA.; ^4^ Institute of High Performance Computing, Agency for Science, Technology and Research, Singapore, Singapore.; ^5^ Programme in Health Services and Systems Research, Duke-NUS Medical School, Singapore, Singapore.; ^6^ Health Services Research Centre, Singapore Health Services, Singapore, Singapore.; ^7^ Department of Emergency Medicine, Singapore General Hospital, Singapore, Singapore.; ^8^ Department of Computing, Imperial College London, London, England, UK.; ^9^ Institute of Data Science, National University of Singapore, Singapore, Singapore.

## Abstract

**Background:** Federated learning (FL) holds promise for safeguarding data privacy in healthcare collaborations. While the term “FL” was originally coined by the engineering community, the statistical field has also developed privacy-preserving algorithms, though these are less recognized. Our goal was to bridge this gap with the first comprehensive comparison of FL frameworks from both domains. **Methods:** We assessed 7 FL frameworks, encompassing both engineering-based and statistical FL algorithms, and compared them against local and centralized modeling of logistic regression and least absolute shrinkage and selection operator (Lasso). Our evaluation utilized both simulated data and real-world emergency department data, focusing on comparing both estimated model coefficients and the performance of model predictions. **Results:** The findings reveal that statistical FL algorithms produce much less biased estimates of model coefficients. Conversely, engineering-based methods can yield models with slightly better prediction performance, occasionally outperforming both centralized and statistical FL models. **Conclusion:** This study underscores the relative strengths and weaknesses of both types of methods, providing recommendations for their selection based on distinct study characteristics. Furthermore, we emphasize the critical need to raise awareness of and integrate these methods into future applications of FL within the healthcare domain.

## Introduction

Privacy regulations, such as the European Union General Data Protection Regulation [1], have introduced substantial challenges to traditional data-sharing strategies in cross-institutional medical research collaborations. Consequently, federated learning (FL) has emerged as a trending solution [2] to address data privacy concerns in the healthcare industry, enabling research collaborations without the necessity of data sharing [3]. FL is a machine learning (ML) paradigm that enables multiple participating sites, referred to as clients, to collaboratively solve a modeling problem without the need to exchange or transfer data [4]. Since its adoption in healthcare, FL has enhanced medical research not only by expanding the scope of research partnerships but also by developing and implementing robust models [3]. For example, during the COVID-19 pandemic, Dayan et al. [5] conducted a study in which they constructed clinical outcome prediction models that outperformed local models by federating data from 20 institutes across the globe.

In the context of clinical FL, an aspect that is often overlooked but is equally critical as prediction tasks is the need to accurately estimate the association between important factors and clinical outcomes, a concept known as “point estimation”, which plays a vital role in guiding the development of interventions and resource allocation strategies. Much like prediction tasks, point estimation tasks can also benefit from FL frameworks by leveraging information from external sources while addressing data privacy concerns. These approaches aim to mitigate bias in point estimates, deviating from the traditional emphasis on optimizing predictive power. For instance, Duan et al. [6] assessed the relative bias of estimated coefficients through simulation studies and used real-world data to showcase their FL algorithm’s comparable performance to pooled analysis when considering estimated odds ratios and their confidence intervals (CIs) for medications associated with fetal loss.

While the engineering community formally introduced the term “FL” [7], the statistical field had been investigating similar privacy-preserving algorithms under different names, such as “distributed learning” [8,9] and “distributed algorithms” [10,11]. However, compared to engineering-based methods, statistics-based FL algorithms have not gained much attention [12] in healthcare research, where ensuring privacy can be of utmost importance.

The fundamental distinction between engineering-based and statistics-based FL algorithms lies in their model agnosticism [12]—whether they can be applied across various types of models or are specific to a particular type. FL algorithms originating from the engineering community typically prioritize predictive power and usually develop model-agnostic FL frameworks, making them versatile for use with different statistical or ML models, including, but not limited to, traditional regression models and various types of neural networks. In contrast, statistics-based methods tend to place greater emphasis on the accuracy of point estimation and, as a result, they are typically developed with a model-specific focus, tailored to meet the decentralized requirements of a single statistical model. This specialization limits their adaptability to other models.

Another distinction lies in the fact that statistics-based methods often prioritize the fundamental task of statistical inference, while engineering-based methods may be primarily designed for prediction tasks. While prediction tasks have become the predominant focus across the various fields employing ML, it is crucial to recognize that healthcare and clinical science also place importance on traditional studies involving nonprediction tasks, especially when using structured data. Beyond predictions, a variety of tasks also require execution within privacy-preserving frameworks. Notable examples include exploring connections between exposures and outcomes [6,13], phenotyping [14,15], and even optimizing individualized treatment [16], all of which employ model-specific [12] FL methods. Since these tasks involve nonpredictive aspects and often require statistical inferences, model-agnostic FL algorithms may not be well suited for these purposes.

There is a need for further exploration to understand how different types of healthcare research centered on structured data may benefit from distinct advantages offered by statistics-based and engineering-based privacy-preserving algorithms [12]. To our knowledge, no empirical comparisons between FL methods from these 2 distinct fields have been reported. This benchmarking study aims to bridge this gap by evaluating FL frameworks from both engineering and statistical domains. Specifically, we apply various FL frameworks on both simulated and real-world data, aiming to evaluate the performance in terms of measuring bias and uncertainty of point estimates, as well as prediction accuracy. We also seek to establish a comprehensive tutorial and recommendations for future researchers.

## Materials and Methods

We evaluated 7 FL frameworks, encompassing 3 statistics-based FL algorithms—Grid binary LOgistic REgression (GLORE) [17], Divide-and-Conquer (DAC) [18], and data-Shielding High-dimensional Integrative Regression (SHIR) [19]—and 4 engineering-based FL algorithms—FedAvg [7], FedAvgM [20], *q*-FedAvg [21], and FedProx [22]—for performing both logistic regression and least absolute shrinkage and selection operator [23] (Lasso) regression. Among these, GLORE, FedAvg, and FedProx are commonly employed [12] in FL studies involving clinical structured data. The other 2 engineering-based FL methods, FedAvgM and *q*-FedAvg, though less frequently applied in clinical settings thus far [12], are notable for their potential in handling nonidentical data distributions and improving fair resource allocation [20,21]. DAC and SHIR were chosen for their shrinkage capabilities in high-dimensional data. A brief introduction to these frameworks is available in Table 1, and detailed technical information can be found in Section A of the Supplementary Materials.

**Table 1.** Overview of benchmark FL frameworks

Framework	Description
GLORE [17]	A model-specific FL algorithm designed for logistic regression, capable of estimating coefficients, variance-covariance matrix, and goodness-of-fit test statistics
DAC [18]	A model-specific screening and one-step linearization infused FL algorithm to fit sparse logistic regression
SHIR [19]	A model-specific data-shielding high-dimensional integrating regression that accommodates heterogeneity in both the covariate distribution and model parameters
FedAvg [7]	The pioneering model-agnostic FL algorithm, where the term “FL” was originally coined
FedAvgM [20]	Built upon FedAvg and enhanced with server momentum [20] to handle nonidentical data [20]
*q*-FedAvg [21]	Built upon FedAvg and enhanced with a novel optimization objective [21] to achieve a more uniform FL model performance across all clients [21]
FedProx [22]	Built upon FedAvg and enhanced to better handle both systems and statistical heterogeneity [7,35] in FL [22]

### Overview

Our evaluation consisted of 2 main phases: first, we assessed the frameworks using simulated data with known ground truth effect sizes for covariates, providing a controlled testing environment; second, we utilized real-world clinical data to evaluate performance of these frameworks in practical, real-world scenarios, particularly for downstream prediction tasks. We conducted simulations using data from 3 client sites referred to as “Site1”, “Site2” and “Site3”, along with a central dataset created by aggregating data from all clients, which we will refer to as “central”. In this way, we independently created local models using the data from each site. We then developed a meta model by pooling the parameters of the local models, weighted by sample size. Finally, we generated a central model by fitting it with the central data. Our simulations covered 3 primary scenarios: data distribution shifts based on mean, data distribution shifts based on variance, and model shifts, as summarized in Table 2.

**Table 2.** Summary of experimental settings

Experiment type	Setting	Details
Simulated data	I	Covariate shifts in mean (*α* = 0.1, 0.2, 0.3, 0.4)
	II	Covariate shifts in standard deviation (*α* = 0.1, 0.2, 0.3, 0.4)
	III	Effect size shifts (*α* = 0.1, 0.2)
Real data	A	Artificial partitioned MIMIC data (homogeneously) with sample size: 3,628 (site 1) and 5,443 (site 2)
	B	Artificial partitioned SGH data (homogeneously), with sample size: 13,789 (site 1), 26,766 (site 2), and 40,555 (site 3)
	C	Artificial partitioned MIMIC data (heterogeneously by age) with sample size: 4,820 (site 1) and 4,251 (site 2)
	D	Artificial partitioned SGH data (heterogeneously by age) with sample size: 22,173 (site 1), 39,597 (site 2), and 19,340 (site 3)
	E	MIMIC data and SGH data with sample size 9,071 (MIMIC) and 81,110 (SGH)

For low-dimensional simulation scenarios, we conducted 2 experiments: one with a small sample size and another with a large sample size, aligning with the respective shifting patterns. The sample size and dimensions were fixed for the high-dimensional scenarios. In addition to using simulated data, we formed cohorts from 2 real-world electronic health records (EHR) datasets: Medical Information Mart for Intensive Care IV Emergency Department (MIMIC-IV-ED) database [24] and the Singapore General Hospital (SGH) emergency department (ED) data [25]. We conducted federations separately for homogeneously and heterogeneously partitioned SGH and MIMIC data. Consistent with the simulated data configuration, we established an equal number of clients for the artificially partitioned datasets, with 3 clients for the SGH data and 2 clients for the MIMIC data, due to the limited size of the study cohorts. Furthermore, we conducted a federation between the MIMIC and SGH datasets, resulting in 2 clients. More specific information about the cohort formation settings is available in Table 2. More details regarding simulation settings are explained in “Simulated data”.

### Simulated data

Data simulation was conducted using R 4.2.1. We considered a total of 3 participating sites that provide a realistic representation for cross-silo FL settings in healthcare, often involving multiple institutions or hospitals. Let *p* denote the total number of predictors, and *s* denote the number of predictors with nonzero effect sizes. In both simulation settings of low and high dimensions, we fixed *s* = 6; *p* = 20 for low dimensions and *p* = 100 for high dimensions. The predictors with nonzero effect size were denoted by *X_i_*, where *i* = 1, 2, 3, …, 6, and their corresponding nonzero effect sizes were denoted as *β_i_*, where *i* = 1, 2, 3, …, 6. We generate binary outcomes as Bernoulli random variables via a logistic regression model: logit{*E*(*y*| *X*) = *X^T^β*}, where logit(*x*) =  log {*x*/(1 − *x*)}. The simulations for both low- and high-dimension scenarios covered 3 distinct settings detailed in Table 2: setting I involved covariate mean shifts, setting II addressed covariate standard deviation shifts, and setting III focused on shifts in effect sizes.

The values of *β_i_* were set as follows: −2, 1, 0.8, 0.4, 0.2, and 0.1 for *i* = 1, 2, 3, …6 throughout settings I and II. In setting III, which accounts for shifts in effect size, the values of *β_i_* were adjusted to (1 − α)*β_i_*, *β_i_*, and (1 + *α*)*β_i_* for site 1, site 2, and site 3, respectively, where α serves as the shifting parameter. Similarly, for settings I and II, corresponding to covariate distribution shifting, the means of covariates were modified to (1 − *α*)*μ_i_*, *μ_i_*, and (1 + *α*)*μ_i_* for site 1, site 2, and site 3, and the standard deviations of covariates were modified to (1 − *α*)σ*_i_*, *σ_i_*, and (1 + *α*)*σ_i_* for site 1, site 2, and site 3.

We conducted all 3 simulation settings for 2 distinct sample size scenarios in the low-dimension experiments: one with relatively small sample sizes, where the sample sizes for site 1, site 2, and site 3 were 1,000, 2,000, and 4,000, respectively; and another with relatively large sample sizes, where the sample sizes for site 1, site 2, and site 3 were 3,000, 6,000, and 12,000, respectively. For the high-dimension experiments, we only used the small sample size scenario, with site 1, site 2, and site 3 having 1,000, 2,000, and 4,000 samples, respectively. The training and testing datasets were partitioned in a 70%-to-30% proportion.

### Real-world datasets

We utilized 2 real ED datasets, MIMIC-IV-ED [24] and EHR from SGH for our experiments with real-world data. The EHR data of SGH was extracted from the SingHealth Electronic Health Intelligence System, and a waiver of consent was granted for EHR data collection and retrospective analysis. The study has been approved by the Singapore Health Services’ Centralized Institutional Review Board, with all data deidentified.

MIMIC is a widely studied [26] open-source dataset, and we follow the data extraction pipelines by Xie et al. [27] This process resulted in the creation of a master dataset, based on which we performed the following cohort formation procedures. Specifically, we formed a cohort of 9,071 samples by filtering the master dataset to include only ED admissions of Asian patients aged 21 and older. We removed observations with missing values in candidate variables, including age, gender, pulse (beats/min), respiration (times/min), peripheral capillary oxygen saturation (SpO_2_; %), diastolic blood pressure (mm Hg), systolic blood pressure (mm Hg), and comorbidities including congestive heart failure, peripheral vascular disease, stroke, dementia, chronic pulmonary disease, and kidney disease. For the SGH dataset, we obtained a subcohort with a total sample size of 81,110 by filtering the original SGH dataset for ED admissions in 2019. We focused on Chinese patients aged 21 and older while eliminating observations with missing values for the same candidate variables. In both datasets, the binary outcome of interest was inpatient mortality.

We designed 5 FL settings based on MIMIC and SGH data as summarized in Table 2. Settings A and B involved independent homogeneous partitioning of MIMIC data and SGH data. Settings C and D entailed independent heterogeneous partitioning of MIMIC data and SGH data based on age. Setting E conducted FL between the full cohorts of MIMIC and SGH data. The sample sizes for each client are provided in Table 2. Detailed descriptive analyses for each client in each setting can be found in Section B of the Supplementary Materials.

### Experiments

The GLORE framework was implemented by adapting the source code available at https://github.com/x1jiang/glore. The DAC algorithm was implemented by adapting the source code available at https://github.com/ChuanHong/solid-copy, and the SHIR algorithm was implemented by adapting the source code available at https://github.com/celehs/SHIR. To implement the FedAvg, FedAvgM, and *q*-FedAvg algorithms, we utilized the Flower [28] framework. As for the FedProx algorithm, we employed the source code from https://github.com/litian96/FedProx. For GLORE, the rounds of iterations were predetermined based on the distance between 2 consecutive *β* values, rather than being determined by user input; additional information is provided in Section A of the Supplementary Materials for reference. Conversely, for the 4 engineering-based frameworks, we determined the rounds of iterations through empirical testing and fine-tuning to identify suitable values for achieving convergence. We also observed that the choice of learning rates had minimal impact on the successful convergence of all engineering-based FL algorithms in the experiments conducted for this study but only on the degree of time efficiency. Further details are available in Section F of the Supplementary Materials.

## Results

### Performance of prediction tasks

**Fig. 1.**
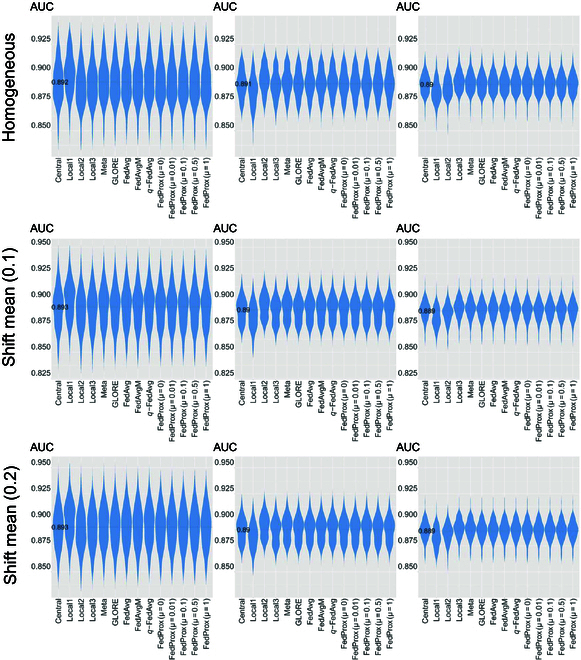
Comparison of prediction performance with a relatively small sample size and mean shifting under a low-dimensional scenario. The performance of both statistics-based and engineering-based FL methods is close to that of the central models. AUC, area under the receiver operating characteristic curve.

The performance of binary outcome prediction is assessed using the receiver operating characteristic (ROC) curve analysis. Figure 1 illustrates model comparisons within the context of Setting I in the simulation studies of the low-dimension scenario, which introduces a shift of covariate means by 10% and 20% while working with a relatively small sample size. The subplots are organized vertically to represent the degree of shifting and horizontally to showcase different testing datasets. Figure 1 reveals that within a single site, no significant differences in prediction task accuracy are observed across all FL frameworks. Additionally, different choices of parameter “*μ*” (proximal term for addressing statistical heterogeneity [22]) for FedProx [22] have minimal impact on the performance of the prediction task. For a more comprehensive view of shifting trends across all settings of both low- and high-dimension scenarios, please refer to Figs. S1 to S9 in Section I.1 of the Supplementary Materials, which shows similar patterns.

Table 3 summarizes the performance of the prediction task using real data in each setting. An intriguing discovery is that while GLORE produces results closely mirroring those of central analysis with coefficients that exhibit remarkable similarity (as demonstrated in Table S7), engineering-based FL models occasionally achieve higher prediction accuracy than centralized analysis, showing significant results in the DeLong ROC test [29]. As shown in Table 3, FedAvg, FedAvgM, and *q*-FedAvg perform significantly better than the central model on the testing data of site 1 obtained from heterogeneously partitioned MIMIC data. Additionally, for the federation between MIMIC and SGH, FedAvg and FedAvgM perform significantly better than the central model on the SGH client.

**Table 3.** Performance assessment of real-world data prediction tasks was measured using AUC values. The DeLong ROC test was conducted for each testing dataset, comparing the central model against each of the other models. Models that performed significantly better than the central model are indicated in bold text.

(A) Prediction performance of federation settings among homogeneously and heterogeneously partitioned MIMIC and SGH data.												
Testing Data			Model									
			Central	Site 1 Local	Site 2 Local	Site 3 Local	Meta	GLORE	FedAvg	FedAvgM	*q*-FedAvg	FedProx
MIMIC	Homogeneous	Site1	0.783 (0.681–885)	0.798 (0.713–0.884)	0.741 (0.625–0.857)		0.799 (0.706–0.891)	0.783 (0.681–0.885)	0.787 (0.687–0.886)	0.787 (0.688–0.886)	0.792 (0.697–0.888)	0.776 (0.673–0.879)
		Site2	0.856 (0.786–0.925)	0.807 (0.724–0.890)	0.849 (0.779–0.919)		0.842 (0.768–0.916)	0.856 (0.786–0.925)	0.863 (0.797–0.930)	0.863 (0.797–0.930)	0.862 (0.795–0.929)	0.858 (0.788–0.928)
		Average	0.820	0.803	0.795		0.821	0.820	0.825	0.825	0.827	0.817
	Heterogeneous	Site1	0.801 (0.686–0.915)	0.773 (0.660–0.886)	0.782 (0.649–0.915)		0.795 (0.681–0.908)	0.801 (0.686–0.915)	**0.821 (0.719–0.922)**	**0.821 (0.719–0.922)**	**0.823 (0.724–0.922)**	0.801 (0.686–0.915)
		Site2	0.793 (0.695–0.891)	0.729 (0.637–0.822)	0.791 (0.684–0.899)		0.782 (0.694–0.871)	0.793 (0.695–0.891)	0.802 (0.707–0.896)	0.802 (0.708–0.896)	0.806 (0.712–0.901)	0.794 (0.696–0.892)
		Average	0.797	0.751	0.787		0.789	0.797	0.812	0.812	0.815	0.798
SGH	Homogeneous	Site1	0.892 (0.860–0.924)	0.887 (0.854–0.919)	0.896 (0.865–0.928)	0.885 (0.852–0.919)	0.892 (0.860–0.924)	0.892 (0.860–0.924)	0.894 (0.862–0.926)	0.894 (0.862–0.926)	0.888 (0.856–0.921)	0.891 (0.859–0.923)
		Site2	0.856 (0.819–0.892)	0.845 (0.807–0.882)	0.854 (0.814–0.894)	0.855 (0.819–0.890)	0.856 (0.819–0.892)	0.856 (0.819–0.892)	0.855 (0.817–0.894)	0.855 (0.817–0.894)	0.847 (0.807–0.886)	0.856 (0.819–0.892)
		Site3	0.884 (0.860–0.909)	0.880 (0.856–0.904)	0.882 (0.856–0.908)	0.881 (0.856–0.906)	0.884 (0.860–0.909)	0.884 (0.860–0.909)	0.884 (0.858–0.909)	0.883 (0.858–0.909)	0.874 (0.847–0.901)	0.884 (0.859–0.908)
		Average	0.877	0.871	0.877	0.874	0.877	0.877	0.878	0.877	0.870	0.877
	Heterogeneous	Site1	0.865 (0.807–0.922)	0.832 (0.764–0.901)	0.853 (0.788–0.918)	0.869 (0.812–0.926)	0.860 (0.801–0.920)	0.865 (0.807–0.922)	0.862 (0.807–0.918)	0.862 (0.807–0.918)	0.856 (0.799–0.913)	0.864 (0.807–0.921)
		Site2	0.860 (0.834–0.886)	0.851 (0.824–0.878)	0.858 (0.832–0.884)	0.851 (0.823–0.878)	0.855 (0.829–0.880)	0.860 (0.834–0.886)	0.862 (0.837–0.887)	0.862 (0.837–0.887)	0.850 (0.824–0.877)	0.860 (0.834–0.886)
		Site3	0.843 (0.808–0.877)	0.828 (0.790–0.865)	0.836 (0.800–0.872)	0.842 (0.808–0.877)	0.838 (0.803–0.872)	0.843 (0.808–0.877)	0.842 (0.807–0.877)	0.842 (0.807–0.877)	0.840 (0.803–0.877)	0.843 (0.808–0.878)
		Average	0.856	0.837	0.849	0.854	0.851	0.856	0.855	0.855	0.849	0.856
(B) Prediction performance of federation between MIMIC and SGH data.												
Testing Data			Model									
			Central	MIMIC Local		SGH Local		GLORE	FedAvg	FedAvgM	*q*-FedAvg	FedProx
MIMIC			0.783 (0.713–0.854)	0.787 (0.718–0.855)		0.780 (0.709–0.852)		0.783 (0.713–0.854)	0.782 (0.711–0.853)	0.782 (0.711–0.853)	0.793 (0.727–0.860)	0.781 (0.710–0.852)
SGH			0.857 (0.837–0.878)	0.830 (0.806–0.853)		0.857 (0.836–0.878)		0.857 (0.837–0.878)	**0.860 (0.840–0.881)**	**0.860 (0.840–0.881)**	0.846 (0.824–0.869)	0.858 (0.837–0.879)
Average			0.820	0.809		0.819		0.820	0.821	0.821	0.820	0.820

### Relative bias of coefficient estimates and CIs

**Fig. 2.**
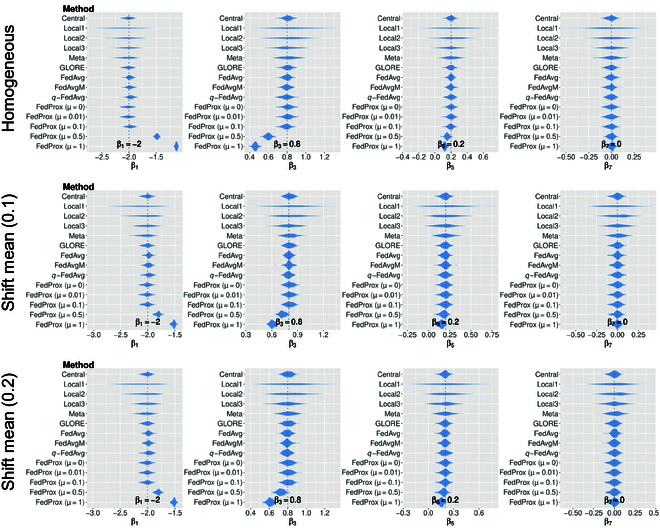
Comparison of estimated coefficients with a relatively small sample size and mean shifting under a low-dimensional scenario.

We use violin plots to visualize the relative bias of coefficient estimates by comparing the model-estimated coefficients to the ground truth values, using 100 simulation runs. As shown in Fig. 2 (representing Setting I with a relatively small sample size), among GLORE, FedAvg, FedAvgM, and *q*-FedAvg, no significant differences are observed in terms of the relative biases of point estimations deviating from the ground truth. However, it is worth noting that the choice of parameter “*μ*” for FedProx results in significantly different levels of bias for point estimations. For a comprehensive view of bias in point estimates across all settings, please refer to Figs. S10 to S15 in Section I.2 of the Supplementary Materials, where similar patterns can be observed.

We additionally conducted 2-sample t-tests to compare the differences in point estimates between the central model and FL algorithms. Table 4 presents the selected testing results for the low-dimensional scenario, and additional details are available in Tables S14 and S15 of the Supplementary Materials. As shown in Table 4, both FedProx and GLORE were tested independently against the central model. The point estimates for both *β*_1_ and *β*_2_ were significantly different between the central model and FedProx under various hyperparameters of FedProx. However, GLORE showed consistency with the central model in both prediction performance (area under the receiver operating characteristic curve [AUC] values) and point estimates.

**Table 4.** Comparison of model pairs regarding both prediction performance and the accuracy of point estimates using a 2-sample *t* test under selected simulation settings of the low-dimensional scenario. The results are exactly the same for both the small and large sample size experiments. Significant results are indicated by “*” (*P* value < 0.05), and nonsignificant results are indicated by “ns”.

Settings	Homogeneous					Shift mean (0.1) SD (0.4)					Shift effect (0.2)				
Model pairs															
Populations	AUC values for each pair of models on the same testing dataset			Point estimates for each pair of models for a single predictor		AUC values for each pair of models on the same testing dataset			Point estimates for each pair of models for a single predictor		AUC values for each pair of models on the same testing dataset			Point estimates for each pair of models for a single predictor	
	Site 1	Site 2	Site 3	*β*_1_	*β*_2_	Site 1	Site 2	Site 3	*β*_1_	*β*_2_	Site 1	Site 2	Site 3	*β*_1_	*β*_2_
Central versus FedProx (*μ* = 1)	ns	ns	ns	*	*	ns	ns	ns	*	*	ns	ns	ns	*	*
GLORE versus FedProx (*μ* = 1)	ns	ns	ns	*	*	ns	ns	ns	*	*	ns	ns	ns	*	*
Central versus FedProx (*μ* = 0.5)	ns	ns	ns	*	*	ns	ns	ns	*	*	ns	ns	ns	*	*
GLORE versus FedProx (*μ* = 0.5)	ns	ns	ns	*	*	ns	ns	ns	*	*	ns	ns	ns	*	*
Central versus GLORE	ns	ns	ns	ns	ns	ns	ns	ns	ns	ns	ns	ns	ns	ns	ns

In addition to direct point estimates, it is important to highlight that only GLORE has the capability to estimate CIs without using bootstrap. We report that the estimated probability of coverage consistently exceeds 90% when using simulated data. For more detailed information, including the average lower and upper bounds of CIs, please refer to Tables S8 to S10 in Section E of the Supplementary Materials. However, as expected, when effect sizes exhibit heterogeneity across sites, GLORE may no longer provide reliable coverage for point estimates at each site. Since the ground truth model (i.e., the conditional distribution *Y* ∣ *X*, where *Y* is the outcome and *X* represents all predictors) is unknown with real data, evaluating the bias of estimated parameters for logistic regression is not feasible.

For the high-dimensional scenario, we primarily compare the true-positive rate (TPR) and positive predictive value (PPV) of variable selection by each FL algorithm against the baselines, which is a key consideration when the parameter vector of interest is sparse [30]. Specifically, TPR represents the percentage of real nonzero coefficients correctly identified as nonzero by the model, and PPV represents the percentage of nonzero coefficients identified by the model that are truly nonzero. As shown in Table 5, the TPR values of all models remain high, especially for engineering-based methods, which are consistently equal to 1 across all settings. However, the PPV for all engineering-based methods remains extremely low (at 0.06) across all settings, suggesting a weak ability for variable selection. In contrast, the 2 statistics-based FL frameworks, DAC and SHIR, exhibit a good balance for both metrics. Notably, the PPV values of both statistics-based FL methods in each setting surpass those of the central models. More details on the comparison of FL frameworks in terms of prediction performance under high-dimensional scenarios can be found in Table S16 and Figs. S7 to S9 in the Supplementary Materials.

**Table 5.** Comparison of FL models and baselines: TPR and PPV values (average of 100 seeds) for variable selection in high-dimensional settings

Settings	Homogeneous		Shift mean (0.1)		Shift mean (0.2)		Shift mean (0.3)		Shift mean (0.4)		Shift mean (0.1) SD (0.1)		Shift mean (0.1) SD (0.2)		Shift mean (0.1) SD (0.3)		Shift mean (0.1) SD (0.4)		Shift effect (0.1)		Shift effect (0.2)	
Model																						
Metrics (100-seed avg.)	TPR	PPV	TPR	PPV	TPR	PPV	TPR	PPV	TPR	PPV	TPR	PPV	TPR	PPV	TPR	PPV	TPR	PPV	TPR	PPV	TPR	PPV
Central	0.987	0.239	0.985	0.228	0.987	0.228	0.990	0.236	0.993	0.229	0.985	0.223	0.983	0.222	0.980	0.218	0.985	0.226	0.988	0.224	0.987	0.224
Local 1	0.842	0.259	0.838	0.261	0.842	0.274	0.835	0.267	0.845	0.261	0.838	0.257	0.813	0.273	0.807	0.273	0.787	0.291	0.828	0.262	0.817	0.268
Local 2	0.912	0.244	0.897	0.239	0.892	0.241	0.897	0.240	0.897	0.241	0.893	0.241	0.892	0.237	0.897	0.244	0.892	0.244	0.903	0.243	0.912	0.240
Local 3	0.957	0.232	0.957	0.230	0.950	0.237	0.953	0.237	0.952	0.245	0.958	0.228	0.963	0.211	0.965	0.221	0.972	0.965	0.218	0.967	0.210
Meta	0.983	0.114	0.985	0.115	0.982	0.118	0.987	0.117	0.985	0.116	0.988	0.115	0.985	0.113	0.988	0.118	0.990	0.119	0.988	0.113	0.988	0.111
DAC	0.848	0.963	0.842	0.958	0.847	0.959	0.850	0.956	0.855	0.959	0.835	0.957	0.835	0.966	0.835	0.963	0.837	0.966	0.852	0.955	0.840	0.945
SHIR	0.795	1.000	0.792	0.998	0.788	0.998	0.802	0.998	0.803	0.998	0.798	0.998	0.807	0.998	0.808	0.998	0.815	1.000	0.807	0.800	1.000
FedAvg	1.000	0.060	1.000	0.060	1.000	0.060	1.000	0.060	1.000	0.060	1.000	0.060	1.000	0.060	1.000	0.060	1.000	0.060	1.000	0.060	1.000	0.060
FedAvgM	1.000	0.060	1.000	0.060	1.000	0.060	1.000	0.060	1.000	0.060	1.000	0.060	1.000	0.060	1.000	0.060	1.000	0.060	1.000	0.060	1.000	0.060
*q*-FedAvg	1.000	0.060	1.000	0.060	1.000	0.060	1.000	0.060	1.000	0.060	1.000	0.060	1.000	0.060	1.000	0.060	1.000	0.060	1.000	0.060	1.000	0.060
FedProx (*μ* = 0)	1.000	0.060	1.000	0.060	1.000	0.060	1.000	0.060	1.000	0.060	1.000	0.060	1.000	0.060	1.000	0.060	1.000	0.060	1.000	0.060	1.000	0.060
FedProx (*μ* = 0.01)	1.000	0.060	1.000	0.060	1.000	0.060	1.000	0.060	1.000	0.060	1.000	0.060	1.000	0.060	1.000	0.060	1.000	0.060	1.000	0.060	1.000	0.060
FedProx (*μ* = 0.1)	1.000	0.060	1.000	0.060	1.000	0.060	1.000	0.060	1.000	0.060	1.000	0.060	1.000	0.060	1.000	0.060	1.000	0.060	1.000	0.060	1.000	0.060
FedProx (*μ* = 0.5)	1.000	0.060	1.000	0.060	1.000	0.060	1.000	0.060	1.000	0.060	1.000	0.060	1.000	0.060	1.000	0.060	1.000	0.060	1.000	0.060	1.000	0.060
FedProx (*μ* = 1)	1.000	0.060	1.000	0.060	1.000	0.060	1.000	0.060	1.000	0.060	1.000	0.060	1.000	0.060	1.000	0.060	1.000	0.060	1.000	0.060	1.000	0.060

### Communication cost

The communication cost of all 7 benchmarked FL methods on simulated data is detailed in Tables S11 and S12. It is evident that GLORE, DAC, and SHIR demonstrate greater communication efficiency when compared to the 4 engineering-based FL methods. SHIR requires only 1 round of communication, whereas DAC has been shown to be effective with predetermined 3 rounds of communication. GLORE consistently required fewer than 6 rounds of communication on average, while all engineering-based FL algorithms necessitated at least 10 rounds for convergence. The communication cost for real data can be found in Table S13. Consistent with the findings observed in the simulation studies, GLORE continues to outperform other methods in terms of communication cost when applied to real data. In parallel, when dealing with real data, FedProx exhibits relatively lower communication efficiency, requiring more rounds of communication for convergence compared to the other 4 methods.

## Discussion

**Fig. 3.**
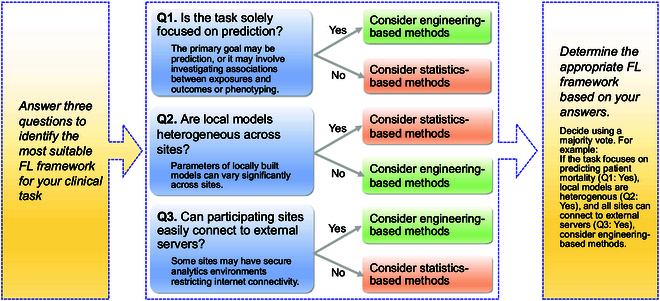
Flowchart illustrating the selection criteria for applying FL algorithms to clinical structured data.

Many studies [31–34] have predominantly focused on evaluating FL approaches within the domain of prediction, while nonprediction tasks such as the estimation of the effects of various factors on outcomes are also important in clinical settings. Lack of guidance for selecting appropriate FL methods for diverse clinical applications underscores the need to discern the varying capabilities of FL methods [12]. We filled this gap by providing practical recommendations for applying FL frameworks to the analysis of clinical structured data, informed by empirical evidence derived from our benchmark study. As illustrated in Fig. 3, we comprehensively discuss and offer suggestions regarding FL frameworks for future clinical research, from perspectives of prediction and nonprediction tasks, data heterogeneity, and real-world implementation challenges.

As highlighted by Li et al. [12], in FL studies primarily concerning unstructured data, prediction performance is often employed as the primary and, most of the time, the sole metric for evaluating the success of FL. However, when dealing with structured data, especially those commonly used in traditional clinical and medical studies, the range of tasks is considerably more diverse compared to those in traditional engineering field. In line with Li et al. [12], we adopt the same categorization to classify clinical decision-making tasks into 2 major categories: prediction and nonprediction tasks. In essence, if the objective is solely to predict an outcome and involves utilizing prediction metrics such as accuracy or those derivable from a confusion matrix, the task falls within the realm of prediction tasks. Conversely, if the goal extends beyond prediction and encompasses tasks such as association studies and phenotyping, it is classified as a nonprediction task.

In the context of nonprediction FL tasks, statistics-based FL frameworks are more suitable than engineering-based approaches in 2 aspects. Firstly, as shown in Tables S8 to S10, statistics-based FL enables convenient estimations of CIs for model parameters, providing users with a straightforward insight into the level of confidence associated with these estimates. In this study, only the GLORE framework allows for the direct calculation of CIs for estimated coefficients in logistic regression without using bootstrap, as detailed in Results. In contrast, engineering-based techniques necessitate additional development to achieve this capability, primarily by resorting to bootstrap, since model-agnostic frameworks do not inherently offer analytical solutions. Nevertheless, it is worth noting that bootstrap can be computationally intensive, which may account for the absence of currently available implementations of engineering methods featuring bootstrap as an option, to the best of our knowledge.

The second reason is rooted in the theoretical soundness of statistics-based methods, often resulting in more accurate parameter estimations and robust hypothesis testing. This is supported by the results in Table S7, where estimations of coefficients by engineering-based methods exhibit more substantial deviations from the centralized model compared to the GLORE model. Similar patterns are evident in the results from simulated data, as illustrated in Table 4 and Tables S14 and S15, where it becomes apparent that variations in hyperparameters can markedly affect the bias of point estimates in engineering-based methods. It is worth noting that hyperparameter tuning in engineering-based methods primarily relies on metrics such as prediction accuracy, as in this study. Consequently, selecting the optimal hyperparameters can be challenging. As shown in Table 4, Fig. 1, and Figs. S1 to S6, the choice of hyperparameters had minimal impact on prediction performance in the corresponding experiments. This makes it difficult for engineering-based FL algorithms to discover less biased point estimates of models through fine-tuning. In light of this empirical evidence, engineering-based methods may introduce more bias than statistics-based methods in nonprediction tasks. Furthermore, these biases may remain undetected in real-world data analysis, where specifying ground truth can be particularly difficult.

Statistics-based and engineering-based FL methods also differ in their approaches to handling data heterogeneity. In the engineering community, statistical heterogeneity in FL is broadly defined as scenarios where the data are not independently and identically distributed (i.i.d.) [35,36]. However, the statistical literature usually distinguishes between heterogeneity in models (conditional distribution of *Y* ∣ *X*) and heterogeneity in covariate distributions (*P*(*X*)), recognizing their distinct impacts on model building and inference [37–39]. A notable example of model heterogeneity can be found in the work by Liu et al. [39], where they propose debiasing distributed Lasso capable of handling both model and covariate heterogeneity. Another example of handling model heterogeneity is available in the work by Gu et al. [38], where they proposed a generalized linear model allowing for population-specific intercepts and *X* coefficients. While these examples contribute to a more thorough understanding of FL data analysis, the complexity of model-specific developments also becomes a notable limitation for statistics-based FL methods, making them more challenging to generalize to different models. For instance, GLORE is limited to handling logistic regression, while engineering based FL solutions can be readily applied to a diverse range of models and clinical research questions [12].

Given the complexity of real-world data, determining the suitability of classic i.i.d.-based FL frameworks without substantial empirical evidence is challenging. Among the 7 FL frameworks benchmarked in this study, both FedAvg and GLORE were theoretically designed only for i.i.d. data. However, both succeeded in handling both heterogeneous simulated and real data in our experiments. Therefore, future researchers may consider benchmarking classic FL frameworks, which assume i.i.d. scenarios, using heterogeneous datasets to further evaluate their effectiveness. However, this strategy may not always yield optimal results, particularly when dealing with “partially-Blackbox” heterogeneity (where data heterogeneity is evident, but model heterogeneity is difficult to specify). As a result, ongoing investigations and evaluations are necessary to thoroughly assess the strengths and weaknesses of different FL methods for handling both data and model heterogeneity.

The engineering-based methods applied to real data in this study have, on occasion, exhibited superior prediction performance, outperforming both the central pooled and local models, as evidenced in Table 3. This pattern consistently remained in both the artificially partitioned SGH/MIMIC data and the federation of MIMIC and SGH data. To comprehend this phenomenon, let us explore the optimization strategies. GLORE employs the Newton–Raphson iteration [17] for FL training, involving second-order approximation [40] of the log-likelihood function by using the Hessian matrix. In contrast, FL frameworks based on FedAvg optimize the target loss function through stochastic gradient descent (SGD). Theoretically, SGD exhibits a slower convergence rate compared to the Newton method, with the latter achieving superior accuracy in reaching the optimal solution [41]. However, SGD provides implicit regularization [42] to the learning model, which leads to improved generalization and prediction performance on test data. Unlike deterministic methods such as the Newton method, SGD tends to converge to wider minima [43], which are associated with potentially better generalization capabilities and robustness on unseen data. As discussed earlier, in the context of nonprediction tasks, this behavior of SGD can be considered disadvantageous compared to model-specific solutions like GLORE, as it may introduce more bias to parameter estimations. However, when the focus shifts to predictive aspects, this behavior appears advantageous, potentially leading to more generalizable prediction models with superior testing accuracy. It is notable that this property of SGD does not always lead to superior prediction performance of FL models. For example, as shown in Table S16 and Fig. S9 of the Supplementary Materials, *q*-FedAvg models perform significantly worse than the central model on the testing dataset of site 3, highlighting the variability in the performance of SGD-based FL methods.

In real-world applications, the implementation of statistics-based FL frameworks and engineering-based FL frameworks can present a notably different level of technical difficulty. For statistics-based methods, iterations usually involve only a few rounds and do not require a central server. FL collaborations, in this case, can be conducted easily as long as participants receive and broadcast informative summary-level statistics to each other. However, for engineering-based methods, which often require at least one central server capable of computation, establishing a secure system with data owners may not be as straightforward as one might imagine. For example, if hospital data can only be accessed via a protected computing system [44] that does not allow connection to outside servers, statistics-based FL methods are more adaptable due to their minimal requirement for the data systems of participating clients.

## Conclusion

In summary, both engineering-based methods and statistics-based FL methods come with their own set of advantages and disadvantages. Our study could provide some valuable empirical insights for future researchers to reference when selecting and adopting these methods. A promising direction for future research involves exploring the fusion of engineering-based and statistics-based FL algorithms to enhance engineering-based methods with statistical inference capabilities while increasing the adaptability of statistics-based methods across a wide range of model types.

## Data Availability

The simulations in this study can be reproduced using the source code available at https://github.com/nliulab/FL-Benchmark. The MIMIC cohort utilized in this study can be obtained from the master dataset generated with the pipelines outlined by Xie et al. [27] for processing the original MIMIC-IV-ED data (https://physionet.org/content/mimic-iv-ed/1.0/) and then by following the details in Results. The SGH data is confidential and not available to the public due to third-party data sharing restrictions.

## Supplementary Materials

Supplementary 1 Sections A to I Figs. S1 to S15 Tables S1 to S19 Algorithms S1 to S7
