# Supplementary material for: Federated Learning in Healthcare: A Benchmark Comparison of Engineering and Statistical Approaches for Structured Data Analysis
Source: Health Data Sci. 2024 Dec 4;4:0196. doi: 10.34133/hds.0196 (PMC11615161; doi:10.34133/hds.0196)
Supplement: Supplementary 1 — Sections A to I Figs. S1 to S15 Tables S1 to S19 Algorithms S1 to S7 [file hds.0196.f1.pdf]

Supplementary Materials of "Federated Learning in Healthcare: A Benchmark Comparison of Engineering and Statistical Approaches for Structured Data Analysis"

A Details of FL Algorithms

A.1 GLORE

The Grid Binary Logistic Regression (GLORE)[1] calculates the traditional LR model in a distributed and privacy-preserving way via Newton-Raphson iteration[2]. For a federation of K-site with n_k records in each site k $(1 \leq k \leq K)$, GLORE calculates the log-likelihood function based on $\sum_{k=1}^{K} n_k$ records is $l(\beta) = \sum_{i=1}^{\sum_{k=1}^{K} n_k} [y_i \log \pi(x_i, \beta) + (1 - y_i) \log(1 - \pi(x_i, \beta))]$, where $x_i = (1, x_{i,1}, ..., x_{i,m})$ for $i = 1, ..., \sum_{k=1}^{K} n_k$.

Algorithm S1 GLORE

Input: $K, \beta^{(0)}, \epsilon$

Client k uses local data to compute $W_k(\bar{X}_k, \beta^{(0)})$ and $\Pi_k(\bar{X}_k, \beta)$ Each client k sends intermediary results θ_k back to the server, and then server aggregates them Server computes $\beta^{(1)}$ and sends back to clients **while** $\|\beta^{(t)} - \beta^{(t-1)}\| \geq \epsilon$ **do**

  Client k uses local data to compute $W_k(\bar{X}_k, \beta^{(t)})$ and $\Pi_k(\bar{X}_k, \beta)$ at t-th iteration Each client k sends intermediary results θ_k back to the server, and then server aggregates them Server computes $\beta^{(t+1)}$ and sends back to clients

end

Specifically, we have

$$\begin{aligned}
\beta^{(t+1)} &= \beta^{(t)} - \left[\frac{\partial^2 l(\beta^{(t)})}{\partial \beta^{(t)} \partial \beta^{(t)\intercal}}\right]^{-1} \frac{\partial l(\beta^{(t)})}{\partial \beta^{(t)}} \\
&= \beta^{(t)} + \left[\bar{X}^\intercal W(\bar{X}, \beta^{(t)}) \bar{X}\right]^{-1} \bar{X}^\intercal \left[\bar{Y} - \Pi(\bar{X}, \beta^{(t)})\right] \\
&= \beta^{(t)} + \left[\sum_{k=1}^{K} \bar{X}_k^\intercal W_k(\bar{X}_k, \beta^{(t)}) \bar{X}_k\right]^{-1} \left\{\sum_{k=1}^{K} \bar{X}_k^\intercal \left[\bar{Y}_k - \Pi_k(\bar{X}_k, \beta)\right]\right\},
\end{aligned}$$

$$W_k(\bar{X}_k, \beta) = \begin{bmatrix} \pi(x_{\sum_{j=1}^{k-1} n_j + 1}, \beta)(1 - \pi(x_{\sum_{j=1}^{k-1} n_j + 1}, \beta)) & \cdots & 0 \\ \vdots & \ddots & \vdots \\ 0 & \cdots & \pi(x_{\sum_{j=1}^{k} n_j}, \beta)(1 - \pi(x_{\sum_{j=1}^{k} n_j}, \beta)) \end{bmatrix},$$

$$\Pi_k(\bar{X}_k, \beta) = \begin{bmatrix} \pi(x_{\sum_{j=1}^{k-1} n_j + 1}, \beta) \\ \vdots \\ \pi(x_{\sum_{j=1}^{k} n_j}, \beta) \end{bmatrix},$$

$$\bar{X} = \begin{bmatrix} \bar{X}_1 \\ \vdots \\ \bar{X}_K \end{bmatrix}, \bar{X}_k = \begin{bmatrix} x_{\sum_{j=1}^{k-1} n_j + 1} \\ \vdots \\ x_{\sum_{j=1}^{k} n_j} \end{bmatrix}, \bar{Y} = \begin{bmatrix} \bar{Y}_1 \\ \vdots \\ \bar{Y}_K \end{bmatrix}, \bar{Y}_k = \begin{bmatrix} y_{\sum_{j=1}^{k-1} n_j + 1} \\ \vdots \\ y_{\sum_{j=1}^{k} n_j} \end{bmatrix} \text{ and } \beta = \begin{bmatrix} \beta_0 \\ \vdots \\ \beta_m \end{bmatrix}$$

A.2 SHIR

SHIR (data-Shielding High-dimensional Integrative Regression)[3] is an innovative estimation procedure designed for sparse regression models that differ across studies. It uses summary-statistics to integrate data while protecting individual datasets, accommodating study heterogeneity, and achieving consistent variable selection with greater statistical efficiency compared to existing distributed methods.

Algorithm S2 SHIR

Input : Observed individual data $\{X^{(m)}, Y^{(m)}\}$ at the mth local site for $m \in [M]$.

for $m \in [M]$, *at the m-th local site* **do**

  Fit $\hat{\beta}^{(m)}_{\text{LASSO}} = \text{argmin}_{\beta^{(m)}} \hat{\mathcal{L}}_m(\beta^{(m)}) + \lambda_m ||\beta^{(m)}_{-1}||_1$ Calculate $\hat{\mathbb{H}}_m = \nabla^2 \hat{\mathcal{L}}_m(\hat{\beta}^{(m)}_{\text{LASSO}})$ and $\hat{g}_m = \hat{\mathbb{H}}_m \hat{\beta}^{(m)}_{\text{LASSO}} - \nabla \hat{\mathcal{L}}_m(\hat{\beta}^{(m)}_{\text{LASSO}})$. Send the summary statistics $\hat{\mathcal{D}}_m = \{n_m, \hat{\mathbb{H}}_m, \hat{g}_m\}$ to the central node.

end

At the central node, obtain $\hat{\beta}^{(\cdot)}_{\text{SHIR}}$ by minimizing:

$\hat{Q}_{\text{SHIR}}(\beta^{(\cdot)}) = N^{-1} \sum_{m=1}^{M} n_m \{\beta^{(m)\mathsf{T}} \hat{\mathbb{H}}_m \beta^{(m)} - 2\beta^{(m)\mathsf{T}} \hat{g}_m\} + \lambda_\rho(\beta^{(\cdot)})$.

Output: The SHIR estimator $\hat{\beta}^{(\cdot)}_{\text{SHIR}}$

A.3 DAC

DAC is a FL method originally proposed by Hong et al.[4] It is a one-step linearization infused FL algorithm to fit sparse logistic regression to distributed datasets. It has been shown to achieve similar statistical efficiency as the full-sample-based estimator[4].

Algorithm S3 DAC

Screening for an active set of predictors

1. use subset $\mathcal{D}_1$ to obtain a ridge estimator

$$\tilde{\beta}^{\mathsf{rid}}_{\Omega_1} = \text{argmax}_\beta\{\hat{\ell}_{\Omega_1}(\beta) + \lambda_{\Omega_1}\sum_{j=1}^{p}\beta_j^2\} \text{ with } 0 < \lambda_{\Omega_1} = \mathbf{O}(n_{\Omega_1}^{-\frac{1}{2}})$$

2. obtain the one-step linear approximation to $\tilde{\beta}_{\Omega_+}$ as $\tilde{\beta}^{\mathsf{lin},1}_{\Omega_+}$, where

$$\tilde{\beta}^{\mathsf{lin},1}_{\Omega_+} = K^{-1}\sum_{k=1}^{K}\{\tilde{\beta}^{\mathsf{rid}}_{\Omega_1} + \hat{\mathbb{A}}_{\mathsf{DAC}}(\tilde{\beta}^{\mathsf{rid}}_{\Omega_1})^{-1}\hat{\mathbf{U}}_{\Omega_k}(\tilde{\beta}^{\mathsf{rid}}_{\Omega_1})\}$$

$$\hat{\mathbf{U}}_{\mathsf{DAC}}(\beta) = K^{-1}\sum_{k=1}^{K}\hat{\mathbf{U}}_{\Omega_k}(\beta), \text{ and } \hat{\mathbb{A}}_{\mathsf{DAC}}(\beta) = K^{-1}\sum_{k=1}^{K}\hat{\mathbb{A}}_{\Omega_k}(\beta)$$

3. apply the LSA for screening

$$\hat{\beta}_{\mathsf{screen}} = \text{argmin}_\beta\{(\tilde{\beta}^{\mathsf{lin},1}_{\Omega_+} - \beta)^{\mathsf{T}}\hat{\mathbb{A}}_{\mathsf{DAC}}(\tilde{\beta}^{\mathsf{lin},1}_{\Omega_+})(\tilde{\beta}^{\mathsf{lin},1}_{\Omega_+} - \beta) + \lambda_{\mathsf{screen}}\sum_{j=1}^{p}\frac{|\beta_j|}{|\tilde{\beta}^{\mathsf{lin},1}_{\Omega_+,j}|^\gamma}\}$$

4. screen for an active set $\mathcal{A}$

$$\hat{\mathcal{A}} = \{j : \hat{\beta}_{\mathsf{screen},j} \neq 0\},$$

   and obtain $\hat{\beta}^{\odot\hat{\mathcal{A}}}_{\mathsf{screen}} = \hat{\beta}_{\mathsf{screen}} \odot \mathbf{I}(\hat{\mathcal{A}})$, where $\mathbf{I}(\hat{\mathcal{A}}) = [1, I(1 \in \hat{\mathcal{A}}), \cdots, I(p \in \hat{\mathcal{A}})]^{\mathsf{T}}$ and $\odot$ indicates elementwise product.

Constructing a linearized adaptive LASSO estimator with the active set

1. obtain the DAC approximated initial estimator, $\tilde{\beta}_{\mathsf{DAC}} = \tilde{\beta}^{[M]}_{\mathsf{DAC}}$, where $\tilde{\beta}^{[M]}_{\mathsf{DAC}}$ is obtained iteratively by letting $\tilde{\beta}^{[0]}_{\mathsf{DAC}} = \hat{\beta}^{\odot\hat{\mathcal{A}}}_{\mathsf{screen}}$,

$$\tilde{\beta}^{[m]}_{\mathsf{DAC}} = \tilde{\beta}^{[m-1]}_{\mathsf{DAC}} + \hat{\mathbb{I}}^{\odot\hat{\mathcal{A}}}_{\mathsf{DAC}}(\tilde{\beta}^{[m-1]}_{\mathsf{DAC}})\hat{\mathbf{U}}_{\mathsf{DAC}}(\tilde{\beta}^{[m-1]}_{\mathsf{DAC}}), \text{ for} m = 1, ..., M$$

   where $\hat{\mathbb{I}}^{[i]\odot\hat{\mathcal{A}}}_{\mathsf{DAC}}(\beta)$ is the $(p + 1) \times (p + 1)$ matrix whose submatrix corresponding to $\hat{\mathcal{A}}$ is $\hat{\mathbb{A}}^{\hat{\mathcal{A}}}_{\mathsf{DAC}}(\beta)^{-1}$ and all other elements are 0;

2. obtain the final DAC estimator as

$$\hat{\beta}_{\mathsf{DAC}} = \text{argmin}_\beta\{\frac{1}{2}\{\tilde{\beta}^{\hat{\mathcal{A}}}_{\mathsf{DAC}} - \beta^{\hat{\mathcal{A}}}\}^{\mathsf{T}}\hat{\mathbb{A}}^{\hat{\mathcal{A}}}_{\mathsf{DAC}}(\tilde{\beta}_{\mathsf{DAC}})(\tilde{\beta}^{\hat{\mathcal{A}}}_{\mathsf{DAC}} - \beta^{\hat{\mathcal{A}}}) + \lambda^{\hat{\mathcal{A}}}_{\Omega_+}\sum_{j=1}^{p}\frac{|\beta_j|}{|\tilde{\beta}_{\mathsf{DAC},j}|^\gamma}\}.$$

Table S1: Notation list of parameters for the four engineering-based FL algorithms

Symbol	Definition	FedAvg	FedAvgM	q-FedAvg	FedProx
K	Total number of clients	√	√	√	√
k	Clients index	√	√	√	√
B	Local minibatch size	√	√	√	√
E	Local epochs	√	√	√	√
η	Learning rate	√	√	√	√
C	The fraction of clients that perform computation on each round	√	√		
w	Model parameters	√	√	√	√
$\mathcal{P}_k$	The set of indexes of data points on client k	√	√		
n_k	The number of the elements in $\mathcal{P}_k$	√	√		
T	Total communication rounds	√	√	√	√
p_k	Probability of device k being selected			√	√

A.4 FedAvg

FedAvg[5] trains and updates models through interaction between a server and multiple clients. Three key parameters (C, E and B) are used to control the amount of computations. On each round, the server selects a C-fraction of clients and the gradient of the loss is calculated on the client using local data. The global batch size is decided by C ($C = 1$ means non-stochastic gradient descent).

Algorithm S4 FedAvg

```
Server executes :
initialize w_0, T
for each round t = 1, 2, ..., T do
    m ← max(C · K, 1)  S_t ←(random set of m clients)  for each
     client k ∈ S_t in parallel do
        w^k_{t+1} ←ClientUpdate(k, w_t)
    end
    m_t ← Σ_{k∈S_t} n_k   w_{t+1} ← Σ_{k∈S_t} (n_k/m_t) w^k_{t+1}
end
ClientUpdate(k, w): //Run on client k
𝓑 ←(split 𝓟_k into batches of size B)
for each local epoch i from 1 to E do
    for batch b ∈ 𝓑 do
        w ← w − η ▽ ℓ(w; b)
    end
    return w to server
end
```

A.5 FedAvgM

Based on FedAvg, FedAvgM[6, 7] introduces momentum v and momentum parameter β. In the original algorithm of FedAvg, the weights are updated by $w \leftarrow w - \Delta w$. FedAvgM instead updates the model by calculating

$$v \leftarrow \beta v + \Delta w$$
$$w \leftarrow w - v$$

By introducing momentum, SGD can speed up convergence, improve the stability of the optimisation and dampen the oscillations of parameter updates[6, 7].

Algorithm S5 FedAvgM

Server executes :
Initialize w_0, T, β, $v_t(v_0 = 0)$.
for *each round* $t = 1, 2, ..., T$ **do**
  $m \leftarrow \max(C \cdot K, 1)$
  $S_t \leftarrow$(random set of m clients)
  for *each client* $k \in S_t$ **in parallel do**
    $w_{t+1}^k \leftarrow$ClientUpdate(k, w_t)
  end
  $m_t \leftarrow \sum_{k \in S_t} n_k$
  $\alpha \leftarrow \sum_{k \in S_t} \frac{n_k}{m_t}(w_{t+1}^k - w_t)$
  $v_{t+1} \leftarrow \beta v_t + (1-\beta)\alpha$
  $w_{t+1} \leftarrow w_t + v_{t+1}$
end
ClientUpdate(k, w)**:** //Run on client k
$\mathcal{B} \leftarrow$(split $\mathcal{P}_k$ into batches of size B)
for *each local epoch i from 1 to E* **do**
  for *batch* $b \in \mathcal{B}$ **do**
    $w \leftarrow w - \eta \bigtriangledown \ell(w; b)$
  end
  return w to server
end

A.6 q-FedAvg

Based on FedAvg, q-FedAvg[8] uses a more complicated dynamic weight determined by the Lipschitz constant (L) of the gradient[9].The parameter q can be tuned based on the desired level of fairness (with larger q inducing more fairness). The q-FedAvg is the same to FedAvg when $q = 0$.

Algorithm S6 q-FedAvg

Input: $m, E, B, T, q, \frac{1}{L}, \eta, w^0, p_k, k = 1, ..., K$
for $t = 0, ..., T-1$ **do**
  Sever selects a subset S_t of m devices at random (each device k is chosen with prob. p_k)
  Server sends w^t to all selected devices
  Each selected device k updates w^t for E epochs of SGD on F_k with step-size η to obtain $\bar{w}_k^{t+1}$
  Each selected device k computes:
  $\Delta w_k^t = L(w_k^t - \bar{w}_k^{t+1})$
  $\Delta_k^t = F_k^q(w^t)\Delta w_k^t$
  $h_k^t = qF_k^{q-1}(w^t)\|\Delta w_k^t\|^2 + LF_k^q(w^t)$
  Each selected device k sends Δ_k^t and h_k^t back to the server
  Server updates w^{t+1} as:
  $w^{t+1} = w^t - \frac{\sum_{k \in S_t} \Delta_k^t}{\sum_{k \in S_t} h_k^t}$
end

A.7 FedProx

Based on FedAvg, FedProx[10] improves its stability to data and system heterogeneity[10] by adding a proximal term to the objective function:

$$\min_w h_k(w; w^t) = F_k(w) + \frac{\mu}{2}\|w - w^t\|^2$$

This modification allows the model to reduce the impact of non-IID while tolerating system heterogeneity. The speed of convergence is related to the penalty constant μ in the proximal term. The γ_k^t-inexactness for client k at iteration t is defined as follows:

Definition 1 (γ_k^t-inexact solution)**.** *For a function* $h_k(w; w_t) = F_k(w) + \frac{\mu}{2}\|w - w_t\|^2$*, and* $\gamma \in [0, 1]$*, we say* w^* *is a* γ_k^t*-inexact solution of* $\min_w h_k(w; w_t)$ *if* $\|\nabla h_k(w^*; w_t)\| \leq \gamma_k^t \|\nabla h_k(w_t; w_t)\|$*, where* $\nabla h_k(w; w_t) = \nabla F_k(w) + \mu(w - w_t)$*. Note that a smaller* γ_k^t *corresponds to higher accuracy.*

By adjusting the value of γ, which varies from device to device, FedProx solves the local function imprecisely.

Algorithm S7 FedProx

Input: $m, E, B, T, \mu, \gamma, w^0, N, p_k, k = 1, ..., K$
for $t = 0, ..., T-1$ **do**
  Server selects a subset S_t of m devices at random (each device k is chosen with probability p_k) Server sends w^t to all chosen devices Each chosen device $k \in S_t$ finds a w_k^{t+1} which is a γ_k^t-inexact minimizer of: $w_k^{t+1} \approx \arg\min_w \; h_k(w; w^t) = F_k(w) + \frac{\mu}{2}\|w - w^t\|^2$ Each device $k \in S_t$ sends w_k^{t+1} back to the server Server aggregates the w's as $w^{t+1} = \frac{1}{m}\sum_{k \in S_t} w_k^{t+1}$
end

B Description of the study cohorts for real data analysis

Table S2: Description of the study cohorts for the SGH dataset

**Homogeneous**		**Site 1**		**Site 2**		**Site 3**	
	Overall	Train	Test	Train	Test	Train	Test
Episodes	81110	8273	5516	16060	10706	24333	16222
Age, mean (SD)	60.90 (18.22)	60.90 (18.31)	60.71 (18.35)	60.99 (18.23)	60.85 (18.24)	60.89 (18.16)	60.94 (18.20)
Gender = Female (%)	40848 (50.4)	4180 (50.5)	2783 (50.5)	8091 (50.4)	5358 (50.0)	12307 (50.6)	8129 (50.1)
Pulse, mean (SD)	81.41 (17.04)	81.66 (17.33)	81.53 (17.11)	81.34 (16.91)	81.18 (17.00)	81.39 (17.06)	81.49 (17.00)
Respiration, mean (SD)	17.71 (1.72)	17.71 (1.67)	17.72 (1.78)	17.71 (1.68)	17.70 (1.74)	17.70 (1.66)	17.72 (1.82)
SpO2, mean (SD)	97.69 (4.66)	97.71 (4.69)	97.59 (5.23)	97.64 (5.00)	97.69 (4.58)	97.74 (4.23)	97.70 (4.75)
Diastolic blood pressure, mean (SD)	73.07 (13.83)	73.03 (14.00)	73.05 (13.97)	73.05 (13.81)	73.10 (13.91)	73.08 (13.77)	73.07 (13.76)
Systolic blood pressure, mean (SD)	134.61 (25.21)	134.32 (25.25)	134.59 (25.42)	134.44 (25.04)	134.51 (25.18)	134.81 (25.18)	134.72 (25.34)
Congestive heart failure (%)	3620 (4.5)	380 (4.6)	238 (4.3)	714 (4.4)	489 (4.6)	1066 (4.4)	733 (4.5)
Peripheral vascular disease (%)	1888 (2.3)	178 (2.2)	118 (2.1)	372 (2.3)	259 (2.4)	539 (2.2)	422 (2.6)
Stroke (%)	5483 (6.8)	550 (6.6)	389 (7.1)	1101 (6.9)	725 (6.8)	1586 (6.5)	1132 (7.0)
Dementia (%)	1929 (2.4)	202 (2.4)	120 (2.2)	384 (2.4)	255 (2.4)	580 (2.4)	388 (2.4)
Chronic pulmonary disease (%)	3595 (4.4)	361 (4.4)	233 (4.2)	708 (4.4)	489 (4.6)	1049 (4.3)	755 (4.7)
Kidney disease (%)	9973 (12.3)	1015 (12.3)	668 (12.1)	1959 (12.2)	1387 (13.0)	2955 (12.1)	1989 (12.3)
Inpatient mortality (%)	924 (1.1)	109 (1.3)	70 (1.3)	190 (1.2)	116 (1.1)	264 (1.1)	175 (1.1)
**Heterogeneous**		**Site 1**		**Site 2**		**Site 3**	
	Overall	Train	Test	Train	Test	Train	Test
Episodes	81110	13304	8869	23758	15839	11604	7736
Age, mean (SD)	60.90 (18.22)	49.44 (14.72)	49.64 (14.72)	61.03 (18.22)	60.75 (18.31)	73.97 (11.81)	73.86 (12.21)
Gender = Female (%)	40848 (50.4)	6424 (48.3)	4390 (49.5)	11969 (50.4)	7868 (49.7)	6075 (52.4)	4122 (53.3)
Pulse, mean (SD)	81.41 (17.04)	82.98 (16.58)	82.53 (16.54)	81.31 (16.92)	81.49 (17.13)	79.83 (17.45)	79.94 (17.61)
Respiration, mean (SD)	17.71 (1.72)	17.66 (1.48)	17.64 (1.55)	17.71 (1.73)	17.73 (1.77)	17.74 (1.83)	17.79 (1.93)
SpO2, mean (SD)	97.69 (4.66)	98.07 (3.55)	98.02 (4.38)	97.68 (4.69)	97.69 (4.54)	97.35 (5.28)	97.24 (5.60)
Diastolic blood pressure, mean (SD)	73.07 (13.83)	74.65 (13.62)	74.90 (13.52)	73.06 (13.91)	73.01 (13.82)	71.20 (13.73)	71.21 (13.86)
Systolic blood pressure, mean (SD)	134.61 (25.21)	130.84 (22.86)	131.61 (23.30)	134.63 (25.17)	134.28 (25.38)	139.01 (26.60)	138.61 (27.13)
Congestive heart failure (%)	3620 (4.5)	338 (2.5)	215 (2.4)	1036 (4.4)	706 (4.5)	754 (6.5)	571 (7.4)
Peripheral vascular disease (%)	1888 (2.3)	189 (1.4)	126 (1.4)	563 (2.4)	374 (2.4)	368 (3.2)	268 (3.5)
Stroke (%)	5483 (6.8)	624 (4.7)	467 (5.3)	1608 (6.8)	1047 (6.6)	1034 (8.9)	703 (9.1)
Dementia (%)	1929 (2.4)	78 (0.6)	38 (0.4)	542 (2.3)	406 (2.6)	515 (4.4)	350 (4.5)
Chronic pulmonary disease (%)	3595 (4.4)	380 (2.9)	256 (2.9)	1043 (4.4)	708 (4.5)	721 (6.2)	487 (6.3)
Kidney disease (%)	9973 (12.3)	906 (6.8)	645 (7.3)	2954 (12.4)	1960 (12.4)	2082 (17.9)	1426 (18.4)
Inpatient mortality (%)	924 (1.1)	79 (0.6)	49 (0.6)	258 (1.1)	205 (1.3)	191 (1.6)	142 (1.8)

Table S3: Description of the study cohorts for the MIMIC dataset

**Homogeneous**		**Site 1**		**Site 2**	
	Overall	Train	Test	Train	Test
Episodes	9071	2177	1451	3266	2177
Age, mean (SD)	58.91 (19.94)	59.09 (20.01)	59.09 (20.05)	58.45 (19.89)	59.31 (19.86)
Gender = Female (%)	4171 (46.0)	1161 (53.3)	777 (53.5)	1788 (54.7)	1174 (53.9)
Pulse, mean (SD)	85.20 (18.50)	84.74 (18.06)	86.12 (19.36)	85.14 (18.33)	85.15 (18.61)
Respiration, mean (SD)	17.63 (2.65)	17.66 (2.55)	17.64 (2.65)	17.58 (2.52)	17.64 (2.94)
SpO2, mean (SD)	98.35 (2.72)	98.26 (3.47)	98.26 (3.51)	98.45 (1.92)	98.37 (2.22)
Diastolic blood pressure, mean (SD)	76.09 (63.41)	75.48 (22.90)	79.11 (151.00)	75.50 (15.70)	75.57 (25.82)
Systolic blood pressure, mean (SD)	133.77 (25.42)	133.33 (23.54)	134.03 (24.76)	133.56 (27.68)	134.37 (24.08)
Congestive heart failure (%)	869 (9.6)	216 (9.9)	140 (9.6)	318 (9.7)	195 (9.0)
Peripheral vascular disease (%)	412 (4.5)	108 (5.0)	57 (3.9)	133 (4.1)	114 (5.2)
Stroke (%)	559 (6.2)	132 (6.1)	96 (6.6)	182 (5.6)	149 (6.8)
Dementia (%)	187 (2.1)	49 (2.3)	25 (1.7)	69 (2.1)	44 (2.0)
Chronic pulmonary disease (%)	1012 (11.2)	236 (10.8)	162 (11.2)	363 (11.1)	251 (11.5)
Kidney disease (%)	215 (2.4)	284 (13.0)	182 (12.5)	396 (12.1)	299 (13.7)
Inpatient mortality (%)	131 (1.4)	37 (1.7)	29 (2.0)	36 (1.1)	29 (1.3)
**Heterogeneous**		**Site 1**		**Site 2**	
	Overall	Train	Test	Train	Test
Episodes	9071	2892	1928	2551	1700
Age, mean (SD)	58.91 (19.94)	52.95 (18.73)	52.28 (18.47)	66.22 (18.92)	65.61 (19.12)
Gender = Female (%)	4171 (46.0)	1523 (52.7)	1073 (55.7)	1381 (54.1)	923 (54.3)
Pulse, mean (SD)	85.20 (18.50)	86.63 (18.82)	85.80 (18.17)	84.21 (18.05)	83.61 (18.81)
Respiration, mean (SD)	17.63 (2.65)	17.54 (2.37)	17.51 (2.43)	17.73 (2.79)	17.74 (3.08)
SpO2, mean (SD)	98.35 (2.72)	98.53 (2.93)	98.49 (2.91)	98.16 (2.41)	98.19 (2.51)
Diastolic blood pressure, mean (SD)	76.09 (63.41)	78.34 (107.39)	77.08 (23.26)	74.41 (18.22)	73.64 (26.70)
Systolic blood pressure, mean (SD)	133.77 (25.42)	132.28 (23.23)	131.72 (22.56)	136.29 (29.90)	134.86 (24.34)
Congestive heart failure (%)	869 (9.6)	212 (7.3)	163 (8.5)	296 (11.6)	198 (11.6)
Peripheral vascular disease (%)	412 (4.5)	116 (4.0)	72 (3.7)	141 (5.5)	83 (4.9)
Stroke (%)	559 (6.2)	130 (4.5)	90 (4.7)	200 (7.8)	139 (8.2)
Dementia (%)	187 (2.1)	30 (1.0)	22 (1.1)	84 (3.3)	51 (3.0)
Chronic pulmonary disease (%)	1012 (11.2)	260 (9.0)	176 (9.1)	344 (13.5)	232 (13.6)
Kidney disease (%)	215 (2.4)	293 (10.1)	219 (11.4)	381 (14.9)	268 (15.8)
Inpatient mortality (%)	131 (1.4)	43 (1.5)	24 (1.2)	40 (1.6)	24 (1.4)

C AUPRC values for real data analysis

Table S4: Prediction performance of federation settings among homogeneously and heterogeneously partitioned MIMIC data, measured by AUPRC values.

Testing Data \ Model		Central	Local 1	Local 2	Meta	GLORE	FedAvg	FedAvgM	q-FedAvg	FedProx
Homogeneous	Site 1	0.081	0.074	0.067	0.077	0.081	0.086	0.086	0.088	0.078
	Site 2	0.117	0.074	0.114	0.125	0.117	0.128	0.127	0.119	0.127
	Average	0.099	0.074	0.091	0.101	0.099	0.107	0.107	0.104	0.103
Heterogeneous	Site 1	0.087	0.065	0.086	0.086	0.087	0.111	0.110	0.110	0.090
	Site 2	0.068	0.029	0.061	0.048	0.068	0.064	0.064	0.069	0.066
	Average	0.077	0.047	0.073	0.067	0.077	0.087	0.087	0.090	0.078

Table S5: Prediction performance of federation settings among homogeneously and heterogeneously partitioned SGH data, measured by AUPRC values.

Testing Data \ Model		Central	Local 1	Local 2	Local 3	Meta	GLORE	FedAvg	FedAvgM	q-FedAvg	FedProx
Homogeneous	Site 1	0.112	0.105	0.110	0.111	0.111	0.112	0.112	0.112	0.110	0.112
	Site 2	0.072	0.064	**0.081**	**0.066**	0.073	0.072	**0.077**	**0.077**	0.076	0.072
	Site 3	0.088	0.088	0.091	0.082	0.088	0.088	0.090	0.090	0.088	0.088
	Average	0.091	0.086	0.094	0.086	0.091	0.091	0.093	0.093	0.091	0.090
Heterogeneous	Site 1	0.059	0.057	0.062	0.050	0.063	0.059	0.062	0.062	0.061	0.060
	Site 2	0.078	0.073	0.081	**0.071**	0.074	0.078	0.080	0.080	0.077	0.079
	Site 3	0.103	0.097	0.103	0.097	0.098	0.103	0.105	0.105	0.108	0.102
	Average	0.080	0.076	0.082	0.073	0.078	0.080	0.082	0.082	0.082	0.081

Table S6: Prediction performance of federation between MIMIC and SGH data, measured by AUPRC values.

Testing Data \ Model	Central	MIMIC	SGH	Meta	GLORE	FedAvg	FedAvgM	q-FedAvg	FedProx
MIMIC	0.052	0.050	0.065	0.052	0.052	0.049	0.049	0.052	0.051
SGH	0.085	**0.068**	0.086	0.085	0.085	**0.088**	**0.088**	**0.076**	0.085
Average	0.068	0.059	0.076	0.069	0.068	0.069	0.069	0.064	0.068

D Point estimates of real data analysis

Table S7: Coefficients of logistic regression estimated by all methods using real data

Settings	Variables	Central	Local 1	Local 2	Meta	GLORE	FedAvg	q-FedAvg	FedAvgM	FedProx
A	Age, mean (SD)	0.686	0.604	0.644	0.539	0.686	0.688	0.198	0.687	0.673
	Gender = Female (%)	-0.626	-1.221	-0.133	-0.708	-0.626	-0.608	-0.202	-0.609	-0.520
	Pulse, mean (SD)	0.516	0.432	0.584	0.474	0.516	0.511	0.134	0.514	0.516
	Respiration, mean (SD)	0.050	-0.021	0.080	-0.011	0.050	0.059	0.013	0.058	0.050
	SpO_2, mean (SD)	-0.144	-0.059	-0.374	-0.169	-0.144	-0.221	-0.047	-0.221	-0.225
	Diastolic blood pressure, mean (SD)	-0.141	-0.686	-0.115	-0.402	-0.141	-0.163	-0.040	-0.162	-0.126
	Systolic blood pressure, mean (SD)	-0.262	-0.178	-0.284	-0.385	-0.262	-0.386	-0.105	-0.385	-0.276
	Congestive heart failure (%)	0.352	-0.045	0.687	0.220	0.352	0.267	0.060	0.266	0.426
	Peripheral vascular disease (%)	-0.076	-0.643	0.456	0.180	-0.076	-0.104	-0.039	-0.102	0.039
	Stroke (%)	0.234	0.194	0.413	0.163	0.234	0.210	0.025	0.210	0.264
	Dementia (%)	0.728	1.649	-0.920	0.378	0.728	0.245	0.069	0.242	0.440
	Chronic pulmonary disease (%)	0.127	-0.001	0.172	0.172	0.127	0.107	0.019	0.106	0.119
	Kidney disease (%)	0.312	0.554	0.055	0.387	0.312	0.242	0.048	0.242	0.269

Settings	Variables	Central	Local 1	Local 2	Local 3	Meta	GLORE	FedAvg	q-FedAvg	FedAvgM	FedProx
B	Age, mean (SD)	0.814	0.837	0.765	0.850	0.819	0.814	0.838	0.186	0.839	0.821
	Gender = Female (%)	-0.107	-0.165	-0.108	-0.067	-0.098	-0.107	-0.113	-0.067	-0.112	-0.089
	Pulse, mean (SD)	0.455	0.487	0.479	0.435	0.458	0.455	0.480	0.094	0.481	0.457
	Respiration, mean (SD)	0.206	0.283	0.258	0.146	0.206	0.206	0.205	0.039	0.205	0.201
	SpO_2, mean (SD)	-0.101	-0.078	-0.121	-0.089	-0.098	-0.101	-0.091	-0.018	-0.089	-0.094
	Diastolic blood pressure, mean (SD)	-0.229	-0.419	-0.242	-0.169	-0.235	-0.229	-0.245	-0.059	-0.246	-0.212
	Systolic blood pressure, mean (SD)	-0.439	-0.186	-0.499	-0.471	-0.432	-0.439	-0.477	-0.079	-0.481	-0.453
	Congestive heart failure (%)	0.287	0.356	0.322	0.238	0.285	0.287	0.228	0.046	0.226	0.272
	Peripheral vascular disease (%)	0.707	1.065	0.341	0.782	0.684	0.707	0.676	0.061	0.672	0.693
	Stroke (%)	0.754	0.889	0.866	0.617	0.746	0.754	0.734	0.071	0.735	0.723
	Dementia (%)	0.290	0.621	-0.209	0.432	0.252	0.290	0.060	0.019	0.066	0.292
	Chronic pulmonary disease (%)	0.159	0.066	-0.146	0.364	0.145	0.159	0.004	-0.027	0.002	0.201
	Kidney disease (%)	0.662	0.308	0.624	0.815	0.666	0.662	0.627	0.083	0.624	0.707

Continued on next page

Settings	Variables	Central	Local 1	Local 2	Meta	GLORE	FedAvg	q-FedAvg	FedAvgM	FedProx
C	Age, mean (SD)	0.638	0.563	0.576	0.569	0.638	0.773	0.214	0.779	0.644
	Gender = Female (%)	-0.868	-1.084	-0.628	-0.870	-0.868	-0.633	-0.183	-0.640	-0.860
	Pulse, mean (SD)	0.479	0.516	0.463	0.491	0.479	0.546	0.136	0.544	0.472
	Respiration, mean (SD)	0.131	0.129	0.106	0.118	0.131	0.043	0.004	0.043	0.124
	SpO_2,, mean (SD)	-0.152	-0.160	-0.255	-0.204	-0.152	-0.151	-0.031	-0.149	-0.175
	Diastolic blood pressure, mean (SD)	-0.061	-1.809	0.102	-0.913	-0.061	-0.285	-0.022	-0.274	-0.051
	Systolic blood pressure, mean (SD)	-0.404	-0.148	-0.570	-0.346	-0.404	-0.392	-0.101	-0.394	-0.406
	Congestive heart failure (%)	0.440	-0.316	0.802	0.208	0.440	0.142	0.050	0.139	0.386
	Peripheral vascular disease (%)	-0.694	-0.324	-0.993	-0.638	-0.694	-0.191	-0.020	-0.192	-0.557
	Stroke (%)	0.520	0.608	0.589	0.599	0.520	0.226	0.017	0.220	0.500
	Dementia (%)	1.050	1.903	0.621	1.302	1.050	0.468	0.040	0.455	0.966
	Chronic pulmonary disease (%)	0.135	0.539	-0.274	0.158	0.135	0.128	0.001	0.122	0.136
	Kidney disease (%)	0.147	-1.061	0.818	-0.180	0.147	0.163	0.050	0.164	0.106

Settings	Variables	Central	Local 1	Local 2	Local 3	Meta	GLORE	FedAvg	q-FedAvg	FedAvgM	FedProx
D	Age, mean (SD)	0.604	0.752	0.797	0.366	0.747	0.604	0.762	0.157	0.760	0.661
	Gender = Female (%)	-0.027	0.228	-0.067	-0.092	-0.044	-0.027	-0.086	-0.079	-0.082	-0.050
	Pulse, mean (SD)	0.499	0.607	0.459	0.548	0.473	0.499	0.484	0.092	0.487	0.502
	Respiration, mean (SD)	0.227	0.338	0.244	0.158	0.241	0.227	0.223	0.044	0.225	0.233
	SpO_2, mean (SD)	-0.099	-0.092	-0.085	-0.131	-0.095	-0.099	-0.090	-0.021	-0.091	-0.099
	Diastolic blood pressure, mean (SD)	-0.240	-0.170	-0.243	-0.274	-0.283	-0.240	-0.224	-0.054	-0.224	-0.230
	Systolic blood pressure, mean (SD)	-0.507	-0.418	-0.638	-0.349	-0.481	-0.507	-0.497	-0.076	-0.497	-0.511
	Congestive heart failure (%)	0.306	-0.236	0.394	0.296	0.843	0.306	0.243	0.032	0.237	0.316
	Peripheral vascular disease (%)	0.918	1.737	0.652	0.963	0.223	0.918	0.727	0.053	0.741	0.847
	Stroke (%)	0.751	1.021	0.743	0.553	0.798	0.751	0.763	0.068	0.759	0.740
	Dementia (%)	0.282	-0.457	0.067	0.391	0.855	0.282	0.036	0.017	0.054	0.149
	Chronic pulmonary disease (%)	0.188	-0.725	0.125	0.267	0.022	0.188	-0.011	-0.021	-0.012	0.119
	Kidney disease (%)	0.656	0.735	0.466	0.635	0.392	0.656	0.581	0.083	0.583	0.562

Continued on next page

Settings	Variables	Central	MIMIC	SGH	Meta	GLORE	FedAvg	q-FedAvg	FedAvgM	FedProx
E	Age, mean (SD)	0.784	0.795	0.783	0.784	0.784	0.826	0.196	0.826	0.786
	Gender = Female (%)	-0.213	-0.975	-0.110	-0.197	-0.213	-0.168	-0.118	-0.168	-0.134
	Pulse, mean (SD)	0.525	0.637	0.513	0.525	0.525	0.486	0.105	0.486	0.524
	Respiration, mean (SD)	0.189	0.113	0.203	0.194	0.189	0.197	0.027	0.197	0.193
	SpO_2, mean (SD)	-0.099	-0.111	-0.094	-0.096	-0.099	-0.101	-0.031	-0.101	-0.095
	Diastolic blood pressure, mean (SD)	-0.277	-0.220	-0.286	-0.279	-0.277	-0.242	-0.047	-0.242	-0.281
	Systolic blood pressure, mean (SD)	-0.412	-0.290	-0.437	-0.422	-0.412	-0.465	-0.091	-0.465	-0.425
	Congestive heart failure (%)	0.409	0.197	0.437	0.413	0.409	0.247	0.052	0.247	0.428
	Peripheral vascular disease (%)	0.540	-0.130	0.627	0.551	0.540	0.627	0.015	0.627	0.610
	Stroke (%)	0.661	0.325	0.724	0.684	0.661	0.701	0.054	0.701	0.711
	Dementia (%)	0.061	0.636	0.009	0.072	0.061	0.132	0.039	0.132	0.017
	Chronic pulmonary disease (%)	-0.142	0.076	-0.202	-0.174	-0.142	0.015	-0.002	0.015	-0.194
	Kidney disease (%)	0.498	0.326	0.528	0.508	0.498	0.585	0.071	0.585	0.521

*Hyperparameter: FedAvg ($\eta = 0.1$), FedAvgM ($\eta = 0.1$), q-FedAvg ($\eta = 0.1$), FedProx ($\eta = 0.01, \mu = 0$)

E Point estimate results of GLORE in Simulation Studies

Table S8: Coverage and average confidence intervals estimated by GLORE (setting I)

**Small sample size**									
**Settings**		$\beta_1 = -2$	$\beta_2 = 1$	$\beta_3 = 0.8$	$\beta_4 = 0.4$	$\beta_5 = 0.2$	$\beta_6 = 0.1$	$\beta_7 = 0$	$\beta_8 = 0$
Homogeneous	Coverage	0.970	0.930	0.960	0.930	0.960	0.940	0.960	0.960
	Lower mean	-2.134	0.914	0.719	0.323	0.119	0.021	-0.084	-0.071
	Upper mean	-1.892	1.095	0.891	0.483	0.275	0.176	0.071	0.084
Shift Mean (0.1)	Coverage	0.970	0.970	0.990	0.950	0.930	0.980	0.930	0.950
	Lower mean	-2.129	0.915	0.720	0.320	0.116	0.023	-0.080	-0.075
	Upper mean	-1.889	1.094	0.890	0.478	0.271	0.176	0.073	0.079
Shift Mean (0.2)	Coverage	0.960	0.940	0.980	0.930	0.940	0.950	0.930	0.950
	Lower mean	-2.128	0.914	0.721	0.320	0.118	0.024	-0.079	-0.074
	Upper mean	-1.889	1.092	0.890	0.477	0.271	0.176	0.073	0.078
Shift Mean (0.3)	Coverage	0.940	0.920	0.980	0.940	0.950	0.970	0.900	0.950
	Lower mean	-2.127	0.916	0.723	0.323	0.118	0.024	-0.078	-0.073
	Upper mean	-1.889	1.093	0.891	0.479	0.270	0.175	0.073	0.078
Shift Mean (0.4)	Coverage	0.960	0.940	0.980	0.930	0.970	0.960	0.970	0.950
	Lower mean	-2.126	0.916	0.722	0.325	0.118	0.024	-0.078	-0.073
	Upper mean	-1.888	1.092	0.889	0.480	0.269	0.174	0.072	0.077
**Large sample size**									
**Settings**		$\beta_1 = -2$	$\beta_2 = 1$	$\beta_3 = 0.8$	$\beta_4 = 0.4$	$\beta_5 = 0.2$	$\beta_6 = 0.1$	$\beta_7 = 0$	$\beta_8 = 0$
Homogeneous	Coverage	0.940	0.960	0.950	0.970	0.940	0.950	0.960	0.930
	Lower mean	-2.069	0.944	0.749	0.353	0.151	0.055	-0.044	-0.046
	Upper mean	-1.930	1.048	0.848	0.445	0.241	0.144	0.045	0.043
Shift Mean (0.1)	Coverage	0.950	0.940	0.920	0.950	0.970	0.880	0.960	0.970
	Lower mean	-2.073	0.951	0.752	0.352	0.154	0.056	-0.045	-0.045
	Upper mean	-1.935	1.054	0.850	0.443	0.243	0.144	0.043	0.043
Shift Mean (0.2)	Coverage	0.940	0.950	0.910	0.940	0.960	0.880	0.960	0.980
	Lower mean	-2.073	0.951	0.751	0.353	0.154	0.057	-0.044	-0.045
	Upper mean	-1.935	1.053	0.849	0.443	0.242	0.144	0.043	0.042
Shift Mean (0.3)	Coverage	0.940	0.940	0.890	0.950	0.940	0.890	0.970	0.990
	Lower mean	-2.072	0.950	0.752	0.354	0.154	0.058	-0.044	-0.044
	Upper mean	-1.935	1.052	0.848	0.444	0.241	0.145	0.043	0.043
Shift Mean (0.4)	Coverage	0.930	0.950	0.920	0.950	0.940	0.900	0.970	0.970
	Lower mean	-2.071	0.950	0.753	0.354	0.154	0.058	-0.043	-0.044
	Upper mean	-1.934	1.051	0.849	0.443	0.241	0.144	0.043	0.042

Table S9: Coverage and average confidence intervals estimated by GLORE (setting II)

**Small sample size**									
**Settings**		$\beta_1 = -2$	$\beta_2 = 1$	$\beta_3 = 0.8$	$\beta_4 = 0.4$	$\beta_5 = 0.2$	$\beta_6 = 0.1$	$\beta_7 = 0$	$\beta_8 = 0$
Homogeneous	Coverage	0.970	0.930	0.960	0.930	0.960	0.940	0.960	0.960
	Lower mean	-2.134	0.914	0.719	0.323	0.119	0.021	-0.084	-0.071
	Upper mean	-1.892	1.095	0.891	0.483	0.275	0.176	0.071	0.084
Shift mean (0.1) SD (0.1)	Coverage	0.950	0.960	0.970	0.970	0.960	0.980	0.950	0.920
	Lower mean	-2.131	0.917	0.721	0.322	0.118	0.024	-0.079	-0.071
	Upper mean	-1.891	1.094	0.890	0.478	0.270	0.176	0.072	0.080
Shift mean (0.1) SD (0.2)	Coverage	0.960	0.930	0.970	0.960	0.950	0.980	0.950	0.920
	Lower mean	-2.127	0.917	0.722	0.322	0.117	0.025	-0.079	-0.069
	Upper mean	-1.888	1.093	0.889	0.476	0.268	0.174	0.071	0.080
Shift mean (0.1) SD (0.3)	Coverage	0.940	0.940	0.980	0.960	0.950	0.990	0.960	0.940
	Lower mean	-2.129	0.918	0.722	0.324	0.118	0.026	-0.078	-0.069
	Upper mean	-1.890	1.094	0.888	0.476	0.266	0.174	0.070	0.079
Shift mean (0.1) SD (0.4)	Coverage	0.940	0.940	0.980	0.970	0.970	0.980	0.970	0.950
	Lower mean	-2.130	0.921	0.724	0.323	0.118	0.026	-0.075	-0.069
	Upper mean	-1.890	1.095	0.889	0.475	0.266	0.173	0.071	0.078
**Large sample size**									
**Settings**		$\beta_1 = -2$	$\beta_2 = 1$	$\beta_3 = 0.8$	$\beta_4 = 0.4$	$\beta_5 = 0.2$	$\beta_6 = 0.1$	$\beta_7 = 0$	$\beta_8 = 0$
Homogeneous	Coverage	0.940	0.960	0.950	0.970	0.940	0.950	0.960	0.930
	Lower mean	-2.069	0.944	0.749	0.353	0.151	0.055	-0.044	-0.046
	Upper mean	-1.930	1.048	0.848	0.445	0.241	0.144	0.045	0.043
Shift mean (0.1) SD (0.1)	Coverage	0.960	0.970	0.920	0.940	0.960	0.890	0.980	0.980
	Lower mean	-2.071	0.950	0.753	0.353	0.156	0.056	-0.044	-0.045
	Upper mean	-1.933	1.052	0.850	0.443	0.243	0.143	0.043	0.043
Shift mean (0.1) SD (0.2)	Coverage	0.930	0.960	0.930	0.950	0.940	0.900	0.970	0.970
	Lower mean	-2.070	0.949	0.752	0.354	0.155	0.057	-0.044	-0.044
	Upper mean	-1.932	1.051	0.848	0.442	0.242	0.143	0.042	0.042
Shift mean (0.1) SD (0.3)	Coverage	0.940	0.940	0.940	0.950	0.970	0.900	0.960	0.980
	Lower mean	-2.070	0.949	0.754	0.354	0.155	0.057	-0.042	-0.043
	Upper mean	-1.932	1.050	0.849	0.442	0.241	0.143	0.043	0.042
Shift mean (0.1) SD (0.4)	Coverage	0.970	0.950	0.930	0.950	0.970	0.910	0.950	0.980
	Lower mean	-2.070	0.949	0.753	0.355	0.155	0.058	-0.042	-0.043
	Upper mean	-1.932	1.049	0.848	0.442	0.240	0.142	0.042	0.041

Table S10: Coverage and average confidence intervals estimated by GLORE (setting III)

**Small sample size**									
**Settings**		$\beta_1 = -2$	$\beta_2 = 1$	$\beta_3 = 0.8$	$\beta_4 = 0.4$	$\beta_5 = 0.2$	$\beta_6 = 0.1$	$\beta_7 = 0$	$\beta_8 = 0$
Homogeneous	Coverage	0.970	0.930	0.960	0.930	0.960	0.940	0.960	0.960
	Lower mean	-2.134	0.914	0.719	0.323	0.119	0.021	-0.084	-0.071
	Upper mean	-1.892	1.095	0.891	0.483	0.275	0.176	0.071	0.084
Shift effect (0.1)	Site1 Coverage	0.000	0.120	0.230	0.740	0.940	0.940	0.960	0.940
	Site2 Coverage	0.800	0.900	0.910	0.910	0.960	0.950	0.960	0.940
	Site3 Coverage	0.560	0.800	0.850	0.880	0.960	0.970	0.960	0.940
	Lower mean	-2.210	0.951	0.747	0.336	0.125	0.024	-0.085	-0.072
	Upper mean	-1.960	1.136	0.922	0.498	0.283	0.181	0.072	0.085
Shift effect (0.2)	Site1 Coverage	0.000	0.000	0.000	0.220	0.870	0.900	0.950	0.950
	Site2 Coverage	0.420	0.660	0.800	0.860	0.940	0.950	0.950	0.950
	Site3 Coverage	0.030	0.270	0.420	0.790	0.930	0.910	0.950	0.950
	Lower mean	-2.270	0.977	0.768	0.348	0.130	0.026	-0.084	-0.073
	Upper mean	-2.014	1.164	0.945	0.512	0.289	0.184	0.074	0.085
**Large sample size**									
**Settings**		$\beta_1 = -2$	$\beta_2 = 1$	$\beta_3 = 0.8$	$\beta_4 = 0.4$	$\beta_5 = 0.2$	$\beta_6 = 0.1$	$\beta_7 = 0$	$\beta_8 = 0$
Homogeneous	Coverage	0.940	0.960	0.950	0.970	0.940	0.950	0.960	0.930
	Lower mean	-2.069	0.944	0.749	0.353	0.151	0.055	-0.044	-0.046
	Upper mean	-1.930	1.048	0.848	0.445	0.241	0.144	0.045	0.043
Shift effect (0.1)	Site1 Coverage	0.000	0.000	0.020	0.360	0.840	0.880	0.930	0.930
	Site2 Coverage	0.450	0.760	0.750	0.900	0.920	0.950	0.930	0.930
	Site3 Coverage	0.070	0.280	0.440	0.830	0.920	0.930	0.930	0.930
	Lower mean	-2.148	0.981	0.779	0.367	0.159	0.059	-0.044	-0.049
	Upper mean	-2.005	1.087	0.880	0.460	0.249	0.149	0.046	0.042
Shift effect (0.2)	Site1 Coverage	0.000	0.000	0.000	0.000	0.440	0.760	0.930	0.950
	Site2 Coverage	0.040	0.390	0.480	0.880	0.900	0.930	0.930	0.950
	Site3 Coverage	0.000	0.010	0.010	0.310	0.760	0.910	0.930	0.950
	Lower mean	-2.204	1.008	0.801	0.378	0.165	0.061	-0.046	-0.049
	Upper mean	-2.057	1.116	0.903	0.472	0.257	0.152	0.045	0.042

F Communication Cost

Table S11: Communication cost for low-dimensional simulations

Settings		GLORE		FedAvg		FedAvgM		q-FedAvg		FedProx	
		Small	Large	Small	Large	Small	Large	Small	Large	Small	Large
	Homogeneous	5.59	5.48	10	10	10	10	30	30	10	20
I	Shift mean (0.1)	5.35	5.12	10	10	10	10	30	30	20	20
	Shift mean (0.2)	5.38	5.14	10	10	10	10	30	30	20	20
	Shift mean (0.3)	5.40	5.17	10	10	10	10	30	30	20	20
	Shift mean (0.4)	5.36	5.17	10	10	10	10	30	30	20	20
II	Shift mean (0.1) SD (0.1)	5.58	5.50	10	10	10	10	30	30	20	20
	Shift mean (0.1) SD (0.2)	5.78	5.87	10	10	10	10	30	30	20	20
	Shift mean (0.1) SD (0.3)	5.93	5.99	10	10	10	10	30	30	20	20
	Shift mean (0.1) SD (0.4)	5.98	6.00	10	10	10	10	30	30	20	20
III	Shift effect (0.1)	5.95	5.98	10	10	10	10	30	30	20	20
	Shift effect (0.2)	6.00	6.00	10	10	10	10	30	30	20	20

Table S12: Communication cost for high-dimensional simulations

Setting		FedAvg	FedAvgM	q-FedAvg	FedProx	SHIR	DAC
	Homogeneous	15	15	60	20	1	3
I	Shift mean (0.1)	15	15	60	20	1	3
	Shift mean (0.2)	15	15	60	20	1	3
	Shift mean (0.3)	15	15	60	20	1	3
	Shift mean (0.4)	15	15	60	20	1	3
II	Shift mean (0.1) SD (0.1)	15	15	60	20	1	3
	Shift mean (0.1) SD (0.2)	15	15	60	20	1	3
	Shift mean (0.1) SD (0.3)	15	15	60	20	1	3
	Shift mean (0.1) SD (0.4)	15	15	60	20	1	3
III	Shift effect (0.1)	15	15	60	20	1	3
	Shift effect (0.2)	15	15	60	20	1	3

Table S13: Communication cost for real data analysis

Settings	GLORE	FedAvg	FedAvgM	q-FedAvg	Fedprox
A	8	10	10	30	50
B	8	10	10	30	50
C	8	10	10	30	50
D	8	10	10	30	50
E	8	10	10	30	50

*Except for GLORE, the number of communication rounds for all other FL methods were predetermined based on fine-tuning or empirical knowledge.

G Statistical tests results for simulation studies

Two-sample t-test results for AUROC values and point estimates.

G.1 Low dimension, small sample size

Table S14: Comparison of model pairs regarding both prediction performance and the accuracy of point estimates using a two-sample t-test under simulation settings of low dimension scenario with small sample size. Significant results are indicated by "*"(using a p-value cutoff of 0.05), and non-significant results are indicated by "ns".

Testing Data	**Homogeneous**					**Shift effect (0.1)**					**Shift effect (0.2)**				
	AUROC Test			Coef Test		AUROC Test			Coef Test		AUROC Test			Coef Test	
**Model Pairs**	Site 1	Site 2	Site 3	β_1	β_2	Site 1	Site 2	Site 3	β_1	β_2	Site 1	Site 2	Site 3	β_1	β_2
Central vs GLORE	ns	ns	ns	ns	ns	ns	ns	ns	ns	ns	ns	ns	ns	ns	ns
Central vs FedAvg	ns	ns	ns	*	ns	ns	ns	ns	*	ns	ns	ns	ns	ns	ns
Central vs FedAvgM	ns	ns	ns	*	*	ns	ns	ns	*	ns	ns	ns	ns	ns	ns
Central vs q-FedAvg	ns	ns	ns	*	*	ns	ns	ns	*	*	ns	ns	ns	*	*
Central vs FedProx ($\mu = 0$)	ns	ns	ns	ns	ns	ns	ns	ns	*	*	ns	ns	ns	*	*
Central vs FedProx ($\mu = 0.01$)	ns	ns	ns	ns	ns	ns	ns	ns	*	*	ns	ns	ns	*	*
Central vs FedProx ($\mu = 0.1$)	ns	ns	ns	*	*	ns	ns	ns	ns	ns	ns	ns	ns	*	ns
Central vs FedProx ($\mu = 0.5$)	ns	ns	ns	*	*	ns	ns	ns	*	*	ns	ns	ns	*	*
Central vs FedProx ($\mu = 1$)	ns	ns	ns	*	*	ns	ns	ns	*	*	ns	ns	ns	*	*

Testing Data	**Shift mean (0.1) SD (0.1)**					**Shift mean (0.1) SD (0.2)**					**Shift mean (0.1) SD (0.3)**				
	AUROC Test			Coef Test		AUROC Test			Coef Test		AUROC Test			Coef Test	
**Model Pairs**	Site 1	Site 2	Site 3	β_1	β_2	Site 1	Site 2	Site 3	β_1	β_2	Site 1	Site 2	Site 3	β_1	β_2
Central vs GLORE	ns	ns	ns	ns	ns	ns	ns	ns	ns	ns	ns	ns	ns	ns	ns
Central vs FedAvg	ns	ns	ns	*	*	ns	ns	ns	*	*	ns	ns	ns	*	*
Central vs FedAvgM	ns	ns	ns	*	*	ns	ns	ns	*	ns	ns	ns	ns	*	*
Central vs q-FedAvg	ns	ns	ns	*	*	ns	ns	ns	*	*	ns	ns	ns	*	*
Central vs FedProx ($\mu = 0$)	ns	ns	ns	ns	ns	ns	ns	ns	ns	ns	ns	ns	ns	ns	ns
Central vs FedProx ($\mu = 0.01$)	ns	ns	ns	ns	ns	ns	ns	ns	ns	ns	ns	ns	ns	ns	ns
Central vs FedProx ($\mu = 0.1$)	ns	ns	ns	ns	ns	ns	ns	ns	ns	ns	ns	ns	ns	ns	ns
Central vs FedProx ($\mu = 0.5$)	ns	ns	ns	*	*	ns	ns	ns	*	*	ns	ns	ns	*	*
Central vs FedProx ($\mu = 1$)	ns	ns	ns	*	*	ns	ns	ns	*	*	ns	ns	ns	*	*

Continued on next page

Model Pairs \ Testing Data	**Shift mean (0.1) SD (0.4)**					**Shift Mean (0.1)**					**Shift Mean (0.2)**				
	AUROC Test			Coef Test		AUROC Test			Coef Test		AUROC Test			Coef Test	
	Site 1	Site 2	Site 3	β_1	β_2	Site 1	Site 2	Site 3	β_1	β_2	Site 1	Site 2	Site 3	β_1	β_2
Central vs GLORE	ns	ns	ns	ns	ns	ns	ns	ns	ns	ns	ns	ns	ns	ns	ns
Central vs FedAvg	ns	ns	ns	*	ns	ns	ns	ns	*	*	ns	ns	ns	*	ns
Central vs FedAvgM	ns	ns	ns	*	ns	ns	ns	ns	*	ns	ns	ns	ns	*	ns
Central vs q-FedAvg	ns	ns	ns	*	*	ns	ns	ns	*	*	ns	ns	ns	*	*
Central vs FedProx ($\mu = 0$)	ns	ns	ns	ns	ns	ns	ns	ns	ns	ns	ns	ns	ns	ns	ns
Central vs FedProx ($\mu = 0.01$)	ns	ns	ns	ns	ns	ns	ns	ns	ns	ns	ns	ns	ns	ns	ns
Central vs FedProx ($\mu = 0.1$)	ns	ns	ns	ns	ns	ns	ns	ns	ns	ns	ns	ns	ns	ns	ns
Central vs FedProx ($\mu = 0.5$)	ns	ns	ns	*	*	ns	ns	ns	*	*	ns	ns	ns	*	*
Central vs FedProx ($\mu = 1$)	ns	ns	ns	*	*	ns	ns	ns	*	*	ns	ns	ns	*	*

Model Pairs \ Testing Data	**Shift Mean (0.3)**					**Shift Mean (0.4)**				
	AUROC Test			Coef Test		AUROC Test			Coef Test	
	Site 1	Site 2	Site 3	β_1	β_2	Site 1	Site 2	Site 3	β_1	β_2
Central vs GLORE	ns	ns	ns	ns	ns	ns	ns	ns	ns	ns
Central vs FedAvg	ns	ns	ns	*	ns	ns	ns	ns	*	*
Central vs FedAvgM	ns	ns	ns	*	ns	ns	ns	ns	*	ns
Central vs q-FedAvg	ns	ns	ns	*	*	ns	ns	ns	*	*
Central vs FedProx ($\mu = 0$)	ns	ns	ns	ns	ns	ns	ns	ns	ns	ns
Central vs FedProx ($\mu = 0.01$)	ns	ns	ns	ns	ns	ns	ns	ns	ns	ns
Central vs FedProx ($\mu = 0.1$)	ns	ns	ns	ns	ns	ns	ns	ns	ns	ns
Central vs FedProx ($\mu = 0.5$)	ns	ns	ns	*	*	ns	ns	ns	*	*
Central vs FedProx ($\mu = 1$)	ns	ns	ns	*	*	ns	ns	ns	*	*

G.2 Low dimension, large sample size

Table S15: Comparison of model pairs regarding both prediction performance and the accuracy of point estimates using a two-sample t-test under simulation settings of low dimension scenario with large sample size. Significant results are indicated by “*”(using a p-value cutoff of 0.05), and non-significant results are indicated by “ns”.

**Testing Data** / **Model Pairs**	**Homogeneous**					**Shift effect (0.1)**					**Shift effect (0.2)**				
	AUROC Test			Coef Test		AUROC Test			Coef Test		AUROC Test			Coef Test	
	Site 1	Site 2	Site 3	β_1	β_2	Site 1	Site 2	Site 3	β_1	β_2					
Central vs GLORE	ns	ns	ns	ns	ns	ns	ns	ns	ns	ns	ns	ns	ns	ns	ns
Central vs FedAvg	ns	ns	ns	ns	ns	ns	ns	ns	ns	ns	ns	ns	ns	*	*
Central vs FedAvgM	ns	ns	ns	ns	ns	ns	ns	ns	ns	ns	ns	ns	ns	*	*
Central vs q-FedAvg	ns	ns	ns	ns	ns	ns	ns	ns	*	*	ns	ns	ns	*	*
Central vs FedProx ($\mu = 0$)	ns	ns	ns	ns	ns	ns	ns	ns	*	*	ns	ns	ns	*	*
Central vs FedProx ($\mu = 0.01$)	ns	ns	ns	ns	ns	ns	ns	ns	*	ns	ns	ns	ns	*	*
Central vs FedProx ($\mu = 0.1$)	ns	ns	ns	ns	ns	ns	ns	ns	ns	ns	ns	ns	ns	ns	ns
Central vs FedProx ($\mu = 0.5$)	ns	ns	ns	*	*	ns	ns	ns	*	*	ns	ns	ns	*	*
Central vs FedProx ($\mu = 1$)	ns	ns	ns	*	*	ns	ns	ns	*	*	ns	ns	ns	*	*

**Testing Data** / **Model Pairs**	**Shift mean (0.1) SD (0.1)**					**Shift mean (0.1) SD (0.2)**					**Shift mean (0.1) SD (0.3)**				
	AUROC Test			Coef Test		AUROC Test			Coef Test		AUROC Test			Coef Test	
	Site 1	Site 2	Site 3	β_1	β_2	Site 1	Site 2	Site 3	β_1	β_2					
Central vs GLORE	ns	ns	ns	ns	ns	ns	ns	ns	ns	ns	ns	ns	ns	ns	ns
Central vs FedAvg	ns	ns	ns	ns	ns	ns	ns	ns	ns	ns	ns	ns	ns	ns	ns
Central vs FedAvgM	ns	ns	ns	ns	ns	ns	ns	ns	ns	ns	ns	ns	ns	ns	ns
Central vs q-FedAvg	ns	ns	ns	ns	ns	ns	ns	ns	ns	ns	ns	ns	ns	ns	ns
Central vs FedProx ($\mu = 0$)	ns	ns	ns	ns	ns	ns	ns	ns	ns	ns	ns	ns	ns	ns	ns
Central vs FedProx ($\mu = 0.01$)	ns	ns	ns	ns	ns	ns	ns	ns	ns	ns	ns	ns	ns	ns	ns
Central vs FedProx ($\mu = 0.1$)	ns	ns	ns	ns	ns	ns	ns	ns	ns	ns	ns	ns	ns	ns	ns
Central vs FedProx ($\mu = 0.5$)	ns	ns	ns	*	*	ns	ns	ns	*	*	ns	ns	ns	*	*
Central vs FedProx ($\mu = 1$)	ns	ns	ns	*	*	ns	ns	ns	*	*	ns	ns	ns	*	*

Continued on next page

Testing Data / Model Pairs	Shift mean (0.1) SD (0.4)					Shift Mean (0.1)					Shift Mean (0.2)				
	AUROC Test			Coef Test		AUROC Test			Coef Test		AUROC Test			Coef Test	
	Site 1	Site 2	Site 3	β_1	β_2	Site 1	Site 2	Site 3	β_1	β_2					
Central vs GLORE	ns	ns	ns	ns	ns	ns	ns	ns	ns	ns	ns	ns	ns	ns	ns
Central vs FedAvg	ns	ns	ns	ns	ns	ns	ns	ns	ns	ns	ns	ns	ns	ns	ns
Central vs FedAvgM	ns	ns	ns	ns	ns	ns	ns	ns	ns	ns	ns	ns	ns	ns	ns
Central vs q-FedAvg	ns	ns	ns	ns	ns	ns	ns	ns	ns	ns	ns	ns	ns	ns	ns
Central vs FedProx ($\mu = 0$)	ns	ns	ns	ns	ns	ns	ns	ns	ns	ns	ns	ns	ns	ns	ns
Central vs FedProx ($\mu = 0.01$)	ns	ns	ns	ns	ns	ns	ns	ns	ns	ns	ns	ns	ns	ns	ns
Central vs FedProx ($\mu = 0.1$)	ns	ns	ns	ns	ns	ns	ns	ns	ns	ns	ns	ns	ns	ns	ns
Central vs FedProx ($\mu = 0.5$)	ns	ns	ns	*	*	ns	ns	ns	*	*	ns	ns	ns	*	*
Central vs FedProx ($\mu = 1$)	ns	ns	ns	*	*	ns	ns	ns	*	*	ns	ns	ns	*	*

Testing Data / Model Pairs	Shift Mean (0.3)					Shift Mean (0.4)				
	AUROC Test			Coef Test		AUROC Test			Coef Test	
	Site 1	Site 2	Site 3	β_1	β_2	Site 1	Site 2	Site 3	β_1	β_2
Central vs GLORE	ns	ns	ns	ns	ns	ns	ns	ns	ns	ns
Central vs FedAvg	ns	ns	ns	ns	ns	ns	ns	ns	ns	ns
Central vs FedAvgM	ns	ns	ns	ns	ns	ns	ns	ns	ns	ns
Central vs q-FedAvg	ns	ns	ns	ns	ns	ns	ns	ns	ns	ns
Central vs FedProx ($\mu = 0$)	ns	ns	ns	ns	ns	ns	ns	ns	ns	ns
Central vs FedProx ($\mu = 0.01$)	ns	ns	ns	ns	ns	ns	ns	ns	ns	ns
Central vs FedProx ($\mu = 0.1$)	ns	ns	ns	ns	ns	ns	ns	ns	ns	ns
Central vs FedProx ($\mu = 0.5$)	ns	ns	ns	*	*	ns	ns	ns	*	*
Central vs FedProx ($\mu = 1$)	ns	ns	ns	*	*	ns	ns	ns	*	*

G.3 High Dimension

Table S16: Comparison of model pairs regarding both prediction performance and the accuracy of point estimates using a two-sample t-test under simulation settings of high dimension scenario. Significant results are indicated by "*"(using a p-value cutoff of 0.05), and non-significant results are indicated by "ns".

Setting	Homogeneous			Shift effect (0.1)			Shift effect (0.2)		
Model Pairs	Site 1	Site 2	Site 3	Site 1	Site 2	Site 3	Site 1	Site 2	Site 3
Central vs FedAvg	*	*	*	*	*	*	*	*	*
Central vs FedAvgM	*	*	*	*	*	*	*	*	*
Central vs q-FedAvg	*	ns	*	*	ns	*	*	ns	*
Central vs FedProx (µ = 1)	ns	ns	ns	ns	ns	ns	ns	ns	ns
Central vs FedProx (µ = 0.5)	ns	ns	ns	ns	ns	ns	ns	ns	ns
Central vs FedProx (µ = 0.1)	ns	ns	ns	ns	ns	ns	ns	ns	ns
Central vs FedProx (µ = 0.01)	ns	ns	ns	ns	ns	ns	ns	ns	ns
Central vs FedProx (µ = 0)	ns	ns	ns	ns	ns	ns	ns	ns	ns
Central vs DAC	ns	ns	ns	ns	ns	ns	ns	ns	ns
Central vs SHIR	ns	ns	*	ns	ns	*	ns	ns	*

Setting	Shift Mean (0.1)			Shift Mean (0.2)			Shift Mean (0.3)			Shift Mean (0.4)		
Model Pairs	Site 1	Site 2	Site 3	Site 1	Site 2	Site 3	Site 1	Site 2	Site 3	Site 1	Site 2	Site 3
Central vs FedAvg	*	ns	*	*	ns	ns	*	ns	ns	*	ns	ns
Central vs FedAvgM	*	ns	*	*	ns	ns	*	ns	ns	*	ns	ns
Central vs q-FedAvg	*	*	*	*	*	*	*	*	*	*	*	*
Central vs FedProx (µ = 1)	ns	ns	ns	ns	ns	ns	ns	ns	ns	ns	ns	ns
Central vs FedProx (µ = 0.5)	ns	ns	ns	ns	ns	ns	ns	ns	ns	ns	ns	ns
Central vs FedProx (µ = 0.1)	ns	ns	ns	ns	ns	ns	ns	ns	ns	ns	ns	ns
Central vs FedProx (µ = 0.01)	ns	ns	ns	ns	ns	ns	ns	ns	ns	ns	ns	ns
Central vs FedProx (µ = 0)	ns	ns	ns	ns	ns	ns	ns	ns	ns	ns	ns	ns
Central vs DAC	ns	ns	ns	ns	ns	ns	ns	ns	ns	ns	ns	ns
Central vs SHIR	ns	ns	*	ns	ns	*	ns	ns	*	ns	ns	*

Continued on next page

Setting	Shift mean (0.1) SD (0.1)			Shift mean (0.1) SD (0.2)			Shift mean (0.1) SD (0.3)			Shift mean (0.1) SD (0.4)		
Model Pairs	Site 1	Site 2	Site 3	Site 1	Site 2	Site 3	Site 1	Site 2	Site 3	Site 1	Site 2	Site 3
Central vs FedAvg	*	ns	*	*	ns	ns	*	ns	ns	*	ns	ns
Central vs FedAvgM	*	ns	*	*	ns	ns	*	ns	ns	*	ns	ns
Central vs q-FedAvg	*	*	*	*	*	*	*	*	*	*	*	*
Central vs FedProx ($\mu = 1$)	ns	ns	ns	ns	ns	ns	ns	ns	ns	ns	ns	ns
Central vs FedProx ($\mu = 0.5$)	ns	ns	ns	ns	ns	ns	ns	ns	ns	ns	ns	ns
Central vs FedProx ($\mu = 0.1$)	ns	ns	ns	ns	ns	ns	ns	ns	ns	ns	ns	ns
Central vs FedProx ($\mu = 0.01$)	ns	ns	ns	ns	ns	ns	ns	ns	ns	ns	ns	ns
Central vs FedProx ($\mu = 0$)	ns	ns	ns	ns	ns	ns	ns	ns	ns	ns	ns	ns
Central vs DAC	ns	ns	ns	ns	ns	ns	ns	ns	ns	ns	ns	ns
Central vs SHIR	ns	ns	*	ns	ns	*	ns	ns	*	ns	ns	*

H P values of DeLong ROC test in simulation studies

H.1 Low dimension, small sample size

Table S17: Proportion of simulation replications for each FL model that have AUROC values tested significantly better than central models, using a p-value cutoff of 0.05.

Testing Data / Model	Homogeneous			Shift effect (0.1)			Shift effect (0.2)		
	Site 1	Site 2	Site 3	Site 1	Site 2	Site 3	Site 1	Site 2	Site 3
Local 1	0.22	0.01	0.00	0.21	0.01	0.00	0.21	0.00	0.00
Local 2	0.00	0.22	0.00	0.00	0.23	0.00	0.00	0.21	0.00
Local 3	0.01	0.01	0.29	0.01	0.02	0.25	0.01	0.02	0.28
Meta	0.11	0.14	0.22	0.15	0.14	0.22	0.09	0.14	0.21
GLORE	0.00	0.00	0.00	0.00	0.00	0.00	0.00	0.00	0.00
FedAvg	0.09	0.20	0.21	0.14	0.15	0.25	0.06	0.16	0.37
FedAvgM	0.12	0.20	0.22	0.08	0.24	0.32	0.10	0.13	0.41
q-FedAvg	0.23	0.18	0.12	0.25	0.19	0.15	0.25	0.19	0.18
FedProx ($\mu = 0$)	0.00	0.00	0.04	0.01	0.01	0.04	0.02	0.02	0.03
FedProx ($\mu = 0.01$)	0.00	0.00	0.03	0.01	0.00	0.05	0.02	0.02	0.04
FedProx ($\mu = 0.1$)	0.01	0.02	0.03	0.01	0.01	0.04	0.02	0.02	0.06
FedProx ($\mu = 0.5$)	0.02	0.05	0.04	0.04	0.05	0.05	0.01	0.04	0.05
FedProx ($\mu = 1$)	0.03	0.03	0.02	0.02	0.05	0.03	0.04	0.04	0.04

Continued on next page

Model \ Testing Data	Shift mean (0.1)			Shift mean (0.2)			Shift mean (0.3)			Shift mean (0.4)		
	Site 1	Site 2	Site 3	Site 1	Site 2	Site 3	Site 1	Site 2	Site 3	Site 1	Site 2	Site 3
Local 1	0.21	0.00	0.00	0.26	0.00	0.00	0.28	0.00	0.01	0.27	0.00	0.00
Local 2	0.01	0.25	0.00	0.02	0.28	0.00	0.02	0.28	0.00	0.01	0.29	0.01
Local 3	0.01	0.02	0.31	0.02	0.02	0.31	0.03	0.03	0.33	0.03	0.01	0.26
Meta	0.08	0.12	0.28	0.11	0.10	0.28	0.10	0.12	0.26	0.12	0.11	0.22
GLORE	0.00	0.00	0.00	0.00	0.00	0.00	0.00	0.00	0.00	0.00	0.00	0.00
FedAvg	0.14	0.20	0.39	0.12	0.22	0.41	0.14	0.24	0.32	0.17	0.20	0.25
FedAvgM	0.11	0.12	0.37	0.13	0.16	0.29	0.10	0.17	0.25	0.15	0.14	0.24
q-FedAvg	0.29	0.18	0.21	0.40	0.18	0.17	0.36	0.19	0.21	0.37	0.21	0.17
FedProx ($\mu = 0$)	0.03	0.00	0.08	0.01	0.02	0.04	0.00	0.05	0.03	0.01	0.02	0.02
FedProx ($\mu = 0.01$)	0.03	0.01	0.07	0.02	0.02	0.03	0.00	0.03	0.04	0.01	0.03	0.02
FedProx ($\mu = 0.1$)	0.03	0.01	0.07	0.00	0.01	0.05	0.02	0.02	0.03	0.01	0.01	0.02
FedProx ($\mu = 0.5$)	0.02	0.01	0.02	0.02	0.01	0.02	0.00	0.01	0.03	0.03	0.00	0.05
FedProx ($\mu = 1$)	0.05	0.01	0.03	0.02	0.01	0.02	0.03	0.00	0.01	0.05	0.02	0.04

Model \ Testing Data	Shift mean (0.1) SD (0.1)			Shift mean (0.1) SD (0.2)			Shift mean (0.1) SD (0.3)			Shift mean (0.1) SD (0.4)		
	Site 1	Site 2	Site 3	Site 1	Site 2	Site 3	Site 1	Site 2	Site 3	Site 1	Site 2	Site 3
Local 1	0.25	0.00	0.00	0.21	0.00	0.00	0.24	0.00	0.00	0.21	0.00	0.00
Local 2	0.00	0.26	0.01	0.01	0.25	0.00	0.02	0.24	0.00	0.01	0.24	0.00
Local 3	0.04	0.02	0.30	0.02	0.02	0.34	0.01	0.03	0.35	0.04	0.04	0.33
Meta	0.11	0.17	0.29	0.08	0.13	0.26	0.14	0.12	0.20	0.16	0.13	0.15
GLORE	0.00	0.00	0.00	0.00	0.00	0.00	0.00	0.00	0.00	0.00	0.00	0.00
FedAvg	0.15	0.13	0.28	0.07	0.19	0.31	0.08	0.16	0.24	0.07	0.15	0.32
FedAvgM	0.09	0.16	0.37	0.08	0.20	0.42	0.06	0.11	0.26	0.10	0.13	0.32
q-FedAvg	0.33	0.20	0.19	0.27	0.16	0.20	0.25	0.20	0.13	0.26	0.19	0.11
FedProx ($\mu = 0$)	0.00	0.02	0.04	0.00	0.02	0.06	0.00	0.01	0.04	0.00	0.01	0.03
FedProx ($\mu = 0.01$)	0.01	0.01	0.04	0.00	0.03	0.04	0.01	0.02	0.03	0.01	0.00	0.03
FedProx ($\mu = 0.1$)	0.00	0.05	0.00	0.00	0.02	0.04	0.02	0.03	0.04	0.01	0.00	0.03
FedProx ($\mu = 0.5$)	0.02	0.02	0.02	0.01	0.01	0.04	0.04	0.00	0.03	0.01	0.01	0.06
FedProx ($\mu = 1$)	0.00	0.01	0.04	0.02	0.00	0.04	0.04	0.01	0.04	0.01	0.01	0.05

H.2 Low dimension, large sample size

Table S18: Proportion of simulation replications for each FL model that have AUC values tested significantly better than central models, using a p-value cutoff of 0.05.

Testing Data / Model	Homogeneous			Shift effect (0.1)			Shift effect (0.2)		
	Site 1	Site 2	Site 3	Site 1	Site 2	Site 3	Site 1	Site 2	Site 3
Local 1	0.23	0.00	0.00	0.19	0.00	0.00	0.28	0.00	0.00
Local 2	0.02	0.21	0.00	0.01	0.19	0.00	0.03	0.19	0.00
Local 3	0.07	0.01	0.24	0.05	0.00	0.24	0.06	0.00	0.29
Meta	0.20	0.13	0.19	0.16	0.10	0.21	0.20	0.13	0.20
GLORE	0.00	0.00	0.00	0.00	0.00	0.00	0.00	0.00	0.00
FedAvg	0.19	0.24	0.56	0.17	0.27	0.63	0.21	0.25	0.60
FedAvgM	0.19	0.26	0.55	0.15	0.27	0.63	0.20	0.25	0.59
q-FedAvg	0.48	0.13	0.15	0.40	0.15	0.15	0.42	0.17	0.15
FedProx ($\mu = 0$)	0.05	0.04	0.02	0.03	0.03	0.03	0.01	0.04	0.06
FedProx ($\mu = 0.01$)	0.04	0.05	0.01	0.04	0.03	0.02	0.04	0.03	0.05
FedProx ($\mu = 0.1$)	0.04	0.05	0.03	0.03	0.01	0.02	0.03	0.04	0.02
FedProx ($\mu = 0.5$)	0.06	0.06	0.04	0.06	0.03	0.04	0.03	0.03	0.06
FedProx ($\mu = 1$)	0.04	0.05	0.03	0.04	0.05	0.03	0.05	0.05	0.04

Continued on next page

Model \ Testing Data	Shift mean (0.1)			Shift mean (0.2)			Shift mean (0.3)			Shift mean (0.4)		
	Site 1	Site 2	Site 3	Site 1	Site 2	Site 3	Site 1	Site 2	Site 3	Site 1	Site 2	Site 3
Local 1	0.18	0.00	0.00	0.20	0.00	0.00	0.22	0.00	0.00	0.24	0.00	0.00
Local 2	0.00	0.14	0.00	0.00	0.17	0.01	0.00	0.14	0.00	0.00	0.17	0.00
Local 3	0.02	0.02	0.24	0.05	0.02	0.33	0.03	0.01	0.27	0.04	0.01	0.29
Meta	0.15	0.14	0.22	0.17	0.13	0.21	0.14	0.13	0.20	0.12	0.16	0.26
GLORE	0.00	0.00	0.00	0.00	0.00	0.00	0.00	0.00	0.00	0.00	0.00	0.00
FedAvg	0.19	0.18	0.55	0.23	0.24	0.55	0.20	0.22	0.56	0.16	0.23	0.50
FedAvgM	0.17	0.16	0.41	0.23	0.24	0.53	0.17	0.21	0.50	0.15	0.17	0.43
q-FedAvg	0.42	0.16	0.18	0.43	0.16	0.16	0.47	0.20	0.19	0.42	0.17	0.26
FedProx ($\mu = 0$)	0.01	0.03	0.01	0.02	0.05	0.04	0.02	0.02	0.01	0.00	0.03	0.01
FedProx ($\mu = 0.01$)	0.02	0.04	0.01	0.02	0.04	0.03	0.02	0.03	0.01	0.02	0.04	0.01
FedProx ($\mu = 0.1$)	0.00	0.08	0.01	0.01	0.05	0.04	0.01	0.03	0.01	0.04	0.06	0.01
FedProx ($\mu = 0.5$)	0.03	0.05	0.02	0.03	0.05	0.06	0.04	0.09	0.03	0.03	0.05	0.03
FedProx ($\mu = 1$)	0.01	0.02	0.01	0.02	0.05	0.02	0.06	0.07	0.04	0.04	0.07	0.03

Model \ Testing Data	Shift mean (0.1) SD (0.1)			Shift mean (0.1) SD (0.2)			Shift mean (0.1) SD (0.3)			Shift mean (0.1) SD (0.4)		
	Site 1	Site 2	Site 3	Site 1	Site 2	Site 3	Site 1	Site 2	Site 3	Site 1	Site 2	Site 3
Local 1	0.27	0.00	0.00	0.21	0.00	0.00	0.19	0.00	0.00	0.21	0.00	0.00
Local 2	0.00	0.16	0.00	0.00	0.14	0.00	0.00	0.18	0.00	0.00	0.18	0.00
Local 3	0.03	0.02	0.23	0.04	0.02	0.26	0.00	0.03	0.26	0.03	0.03	0.24
Meta	0.17	0.13	0.20	0.17	0.13	0.15	0.17	0.15	0.14	0.18	0.16	0.11
GLORE	0.00	0.00	0.00	0.00	0.00	0.00	0.00	0.00	0.00	0.00	0.00	0.00
FedAvg	0.25	0.23	0.52	0.22	0.26	0.52	0.22	0.22	0.48	0.24	0.22	0.46
FedAvgM	0.25	0.23	0.53	0.21	0.25	0.55	0.21	0.20	0.46	0.24	0.22	0.44
q-FedAvg	0.49	0.18	0.11	0.44	0.20	0.15	0.42	0.21	0.12	0.41	0.19	0.08
FedProx ($\mu = 0$)	0.05	0.05	0.02	0.01	0.03	0.02	0.02	0.04	0.03	0.03	0.07	0.04
FedProx ($\mu = 0.01$)	0.03	0.05	0.03	0.01	0.02	0.02	0.03	0.05	0.04	0.02	0.07	0.04
FedProx ($\mu = 0.1$)	0.03	0.05	0.03	0.02	0.04	0.02	0.02	0.05	0.03	0.02	0.05	0.02
FedProx ($\mu = 0.5$)	0.02	0.02	0.02	0.03	0.04	0.04	0.02	0.02	0.02	0.02	0.03	0.02
FedProx ($\mu = 1$)	0.02	0.03	0.03	0.01	0.02	0.04	0.02	0.01	0.04	0.01	0.02	0.02

H.3 High dimension

Table S19: Proportion of simulation replications for each FL model that have AUC values tested significantly better than central models, using a p-value cutoff of 0.05.

Testing Data / Model	Homogeneous			Shift effect (0.1)			Shift effect (0.2)		
	Site 1	Site 2	Site 3	Site 1	Site 2	Site 3	Site 1	Site 2	Site 3
Local 1	0.19	0.00	0.00	0.00	0.00	0.00	0.01	0.00	0.00
Local 2	0.00	0.21	0.02	0.00	0.01	0.00	0.02	0.00	0.02
Local 3	0.04	0.02	0.20	0.04	0.03	0.00	0.02	0.02	0.00
Meta	0.01	0.03	0.01	0.01	0.02	0.01	0.02	0.02	0.01
DAC	0.02	0.07	0.07	0.05	0.06	0.04	0.05	0.05	0.06
SHIR	0.00	0.00	0.00	0.00	0.00	0.00	0.00	0.00	0.00
FedAvg	0.86	0.34	0.50	0.77	0.50	0.62	0.67	0.54	0.67
FedAvgM	0.83	0.36	0.55	0.79	0.50	0.58	0.69	0.59	0.65
q-FedAvg	1.00	0.04	0.00	1.00	0.11	0.00	1.00	0.11	0.00
FedProx ($\mu = 0$)	0.01	0.03	0.04	0.01	0.02	0.02	0.03	0.04	0.05
FedProx ($\mu = 0.01$)	0.01	0.03	0.04	0.01	0.02	0.02	0.04	0.04	0.05
FedProx ($\mu = 0.1$)	0.02	0.04	0.06	0.02	0.05	0.07	0.04	0.06	0.09
FedProx ($\mu = 0.5$)	0.06	0.09	0.08	0.04	0.06	0.09	0.03	0.11	0.11
FedProx ($\mu = 1$)	0.04	0.06	0.08	0.06	0.07	0.10	0.05	0.08	0.09

Continued on next page

Model \ Testing Data	Shift mean (0.1)			Shift mean (0.2)			Shift mean (0.3)			Shift mean (0.4)		
	Site 1	Site 2	Site 3	Site 1	Site 2	Site 3	Site 1	Site 2	Site 3	Site 1	Site 2	Site 3
Local 1	0.01	0.00	0.00	0.00	0.01	0.01	0.00	0.00	0.00	0.00	0.00	0.00
Local 2	0.02	0.00	0.00	0.00	0.00	0.00	0.03	0.00	0.00	0.01	0.00	0.00
Local 3	0.01	0.03	0.01	0.01	0.01	0.00	0.02	0.01	0.01	0.01	0.02	0.00
Meta	0.02	0.00	0.01	0.01	0.00	0.00	0.02	0.00	0.00	0.03	0.00	0.01
DAC	0.04	0.02	0.06	0.02	0.03	0.07	0.04	0.04	0.07	0.02	0.04	0.07
SHIR	0.01	0.00	0.00	0.00	0.01	0.00	0.00	0.00	0.00	0.01	0.00	0.00
FedAvg	0.97	0.20	0.11	0.96	0.29	0.17	0.93	0.31	0.21	0.96	0.30	0.25
FedAvgM	0.98	0.20	0.11	0.96	0.29	0.16	0.95	0.31	0.19	0.95	0.31	0.24
q-FedAvg	1.00	0.01	0.00	1.00	0.01	0.00	1.00	0.02	0.00	1.00	0.04	0.00
FedProx ($\mu = 0$)	0.03	0.02	0.01	0.01	0.02	0.00	0.01	0.03	0.02	0.02	0.04	0.00
FedProx ($\mu = 0.01$)	0.03	0.02	0.01	0.01	0.03	0.01	0.02	0.03	0.02	0.02	0.04	0.00
FedProx ($\mu = 0.1$)	0.03	0.02	0.02	0.01	0.02	0.03	0.03	0.04	0.04	0.03	0.03	0.01
FedProx ($\mu = 0.5$)	0.04	0.02	0.04	0.01	0.04	0.06	0.04	0.02	0.06	0.04	0.06	0.06
FedProx ($\mu = 1$)	0.04	0.01	0.05	0.02	0.04	0.05	0.04	0.02	0.06	0.03	0.07	0.05

Model \ Testing Data	Shift mean (0.1) SD (0.1)			Shift mean (0.1) SD (0.2)			Shift mean (0.1) SD (0.3)			Shift mean (0.1) SD (0.4)		
	Site 1	Site 2	Site 3	Site 1	Site 2	Site 3	Site 1	Site 2	Site 3	Site 1	Site 2	Site 3
Local 1	0.01	0.00	0.00	0.01	0.00	0.00	0.01	0.00	0.00	0.00	0.00	0.00
Local 2	0.01	0.01	0.00	0.03	0.00	0.00	0.05	0.00	0.00	0.04	0.00	0.00
Local 3	0.01	0.01	0.05	0.02	0.02	0.01	0.02	0.04	0.01	0.01	0.04	0.02
Meta	0.02	0.00	0.00	0.01	0.00	0.00	0.02	0.00	0.00	0.02	0.00	0.00
DAC	0.06	0.04	0.08	0.05	0.05	0.06	0.04	0.04	0.06	0.04	0.03	0.04
SHIR	0.01	0.00	0.00	0.01	0.00	0.00	0.01	0.00	0.00	0.01	0.00	0.00
FedAvg	0.94	0.14	0.10	0.97	0.24	0.13	0.98	0.29	0.12	0.97	0.28	0.11
FedAvgM	0.95	0.13	0.11	0.96	0.23	0.13	0.98	0.28	0.12	0.97	0.28	0.11
q-FedAvg	1.00	0.01	0.00	1.00	0.02	0.00	1.00	0.02	0.00	1.00	0.04	0.00
FedProx ($\mu = 0$)	0.00	0.01	0.02	0.04	0.00	0.01	0.00	0.00	0.00	0.03	0.00	0.00
FedProx ($\mu = 0.01$)	0.01	0.01	0.03	0.04	0.00	0.02	0.00	0.00	0.01	0.03	0.00	0.00
FedProx ($\mu = 0.1$)	0.02	0.01	0.03	0.04	0.00	0.02	0.01	0.01	0.03	0.04	0.04	0.02
FedProx ($\mu = 0.5$)	0.02	0.01	0.06	0.03	0.01	0.04	0.02	0.03	0.03	0.04	0.05	0.05
FedProx ($\mu = 1$)	0.02	0.01	0.07	0.03	0.02	0.06	0.02	0.03	0.05	0.05	0.06	0.06

I Plots for simulation studies

I.1 Prediction tasks

I.1.1 Low Dimension

Figure S1: FL Model comparisons by prediction performance under shifting of covariate mean with relatively small sample size.

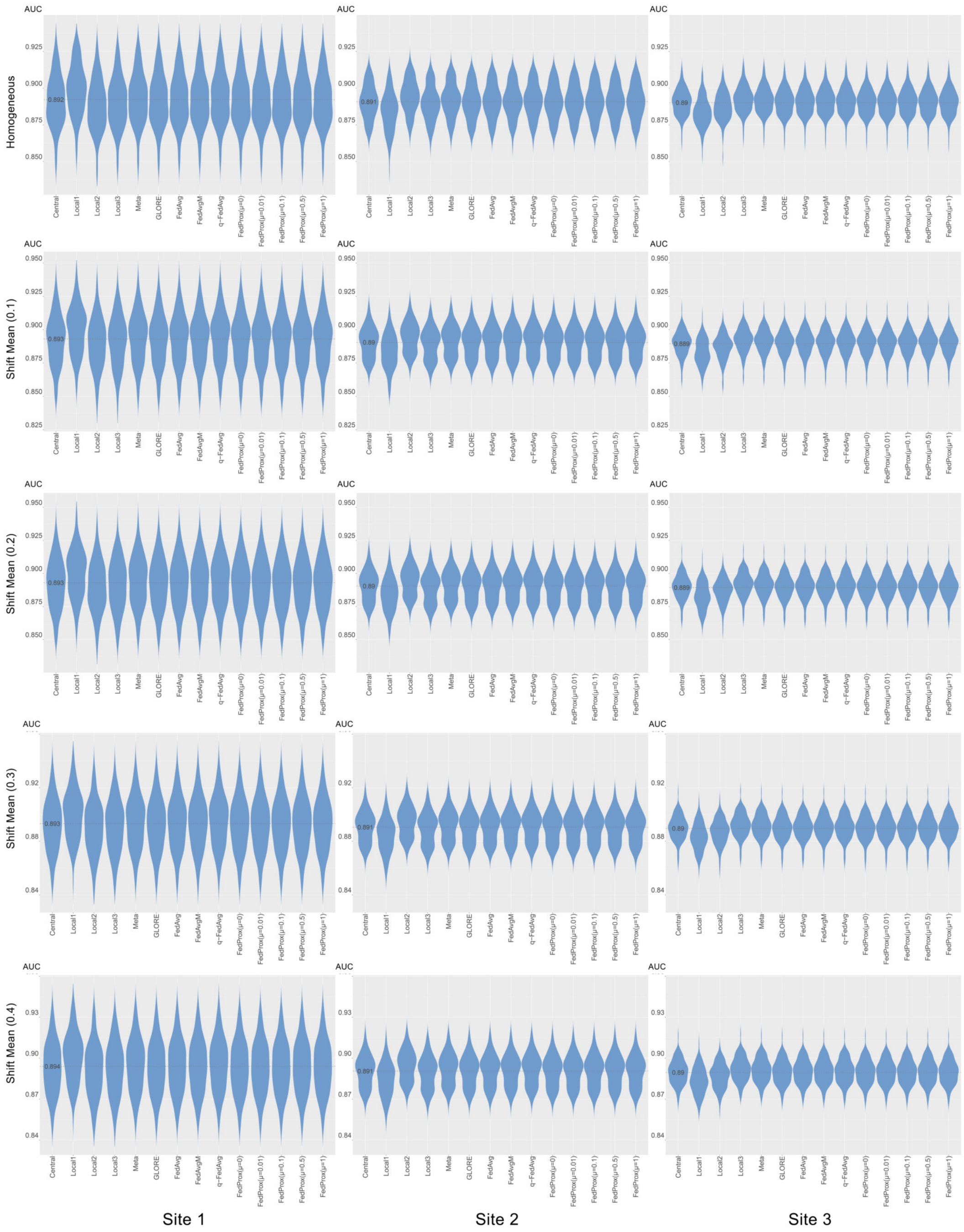

Figure S2: FL Model comparisons by prediction performance under shifting of covariate mean with relatively large sample size.

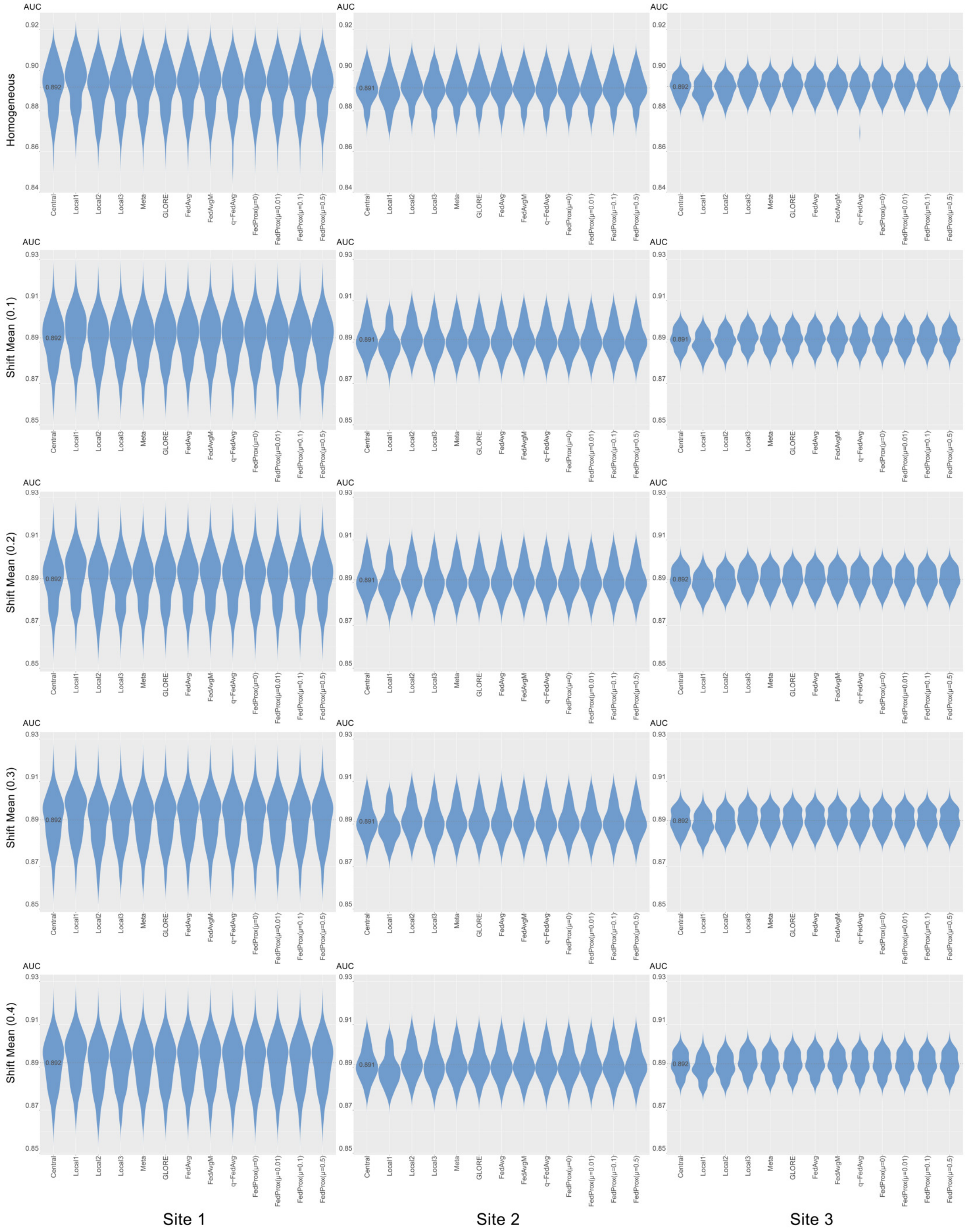

Figure S3: FL Model comparisons by prediction performance under shifting of covariate standard deviation (SD) with relatively small sample size.

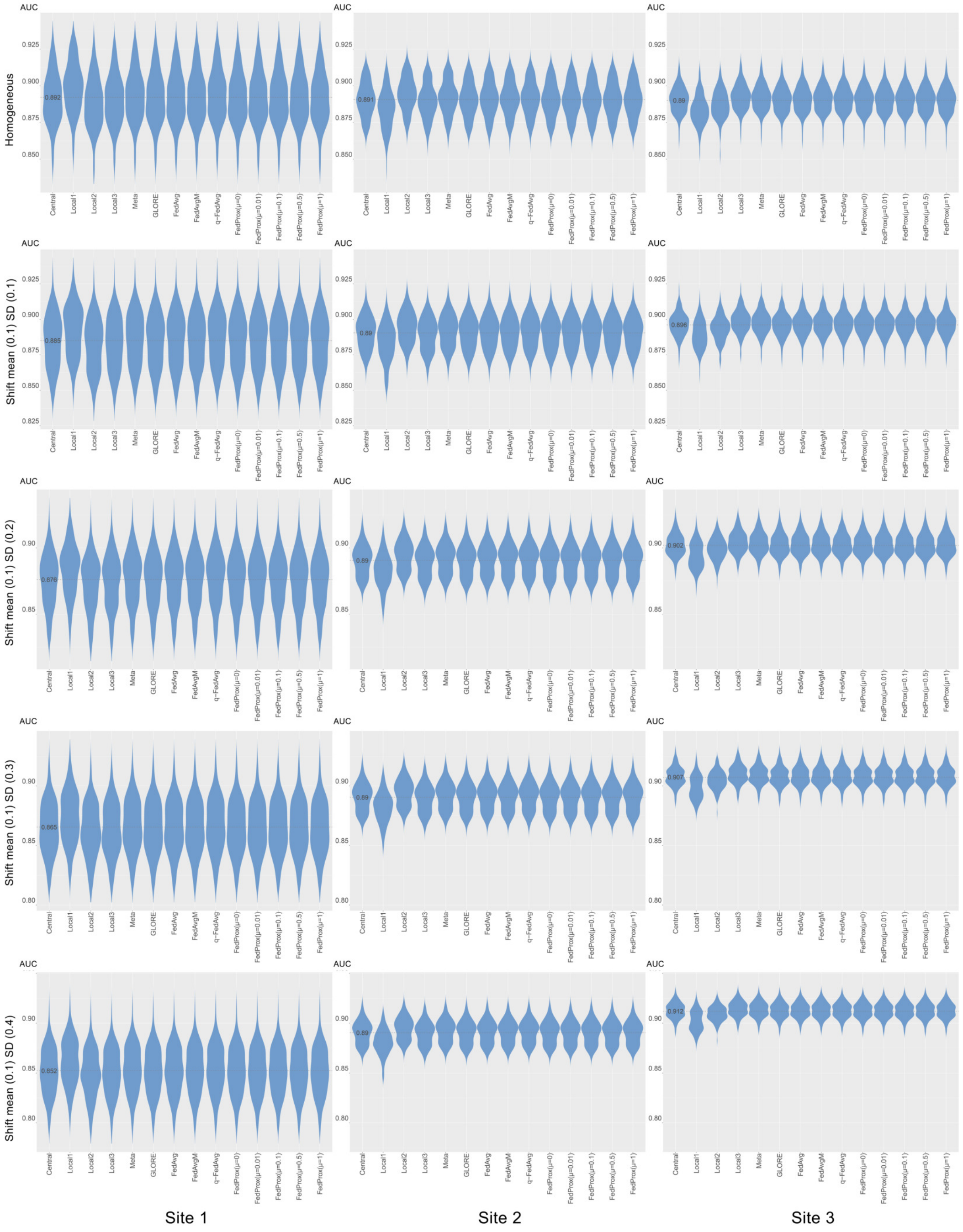

Figure S4: FL Model comparisons by prediction performance under shifting of covariate SD with relatively large sample size.

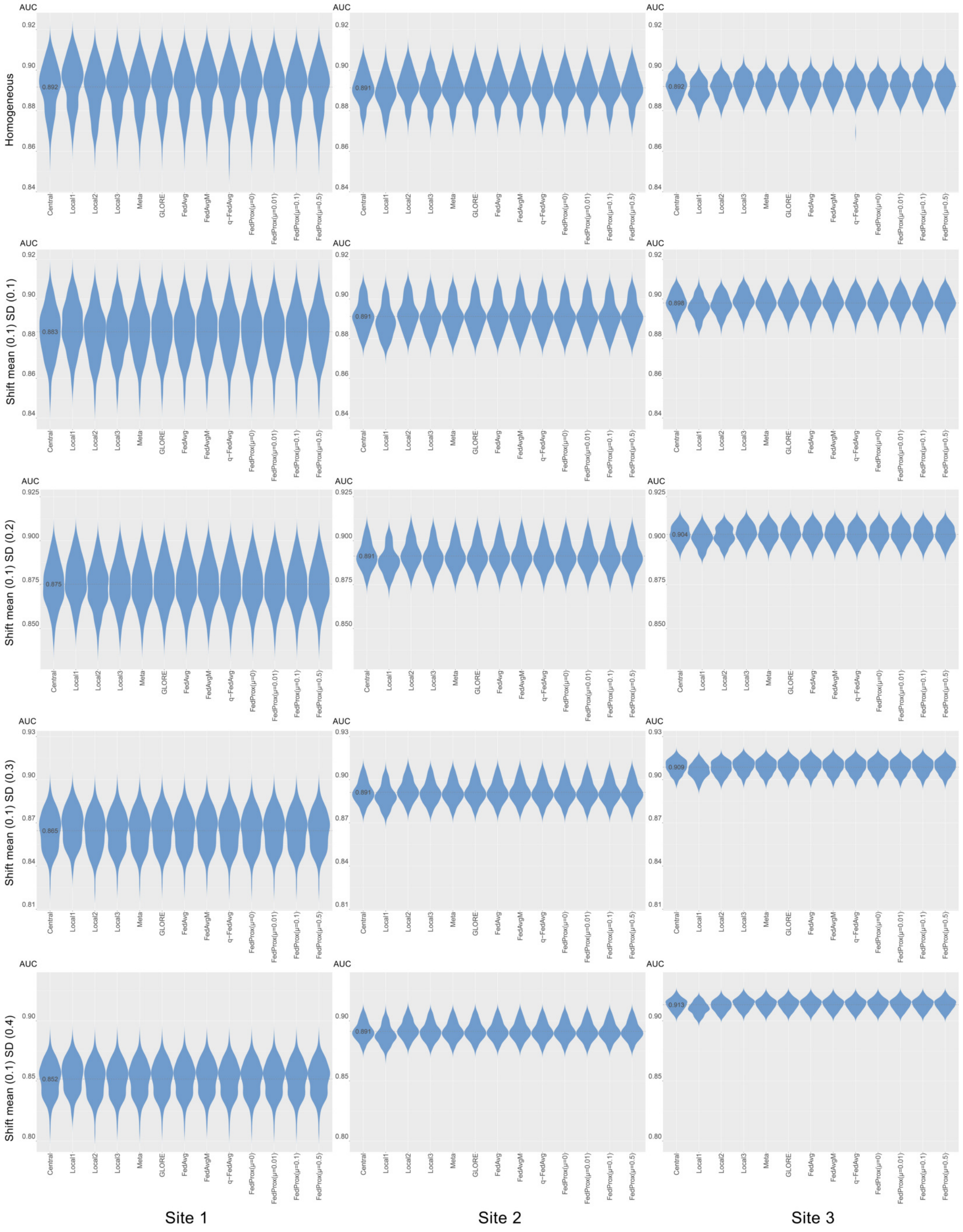

Figure S5: FL Model comparisons by prediction performance under shifting of effect size with relatively small sample size.

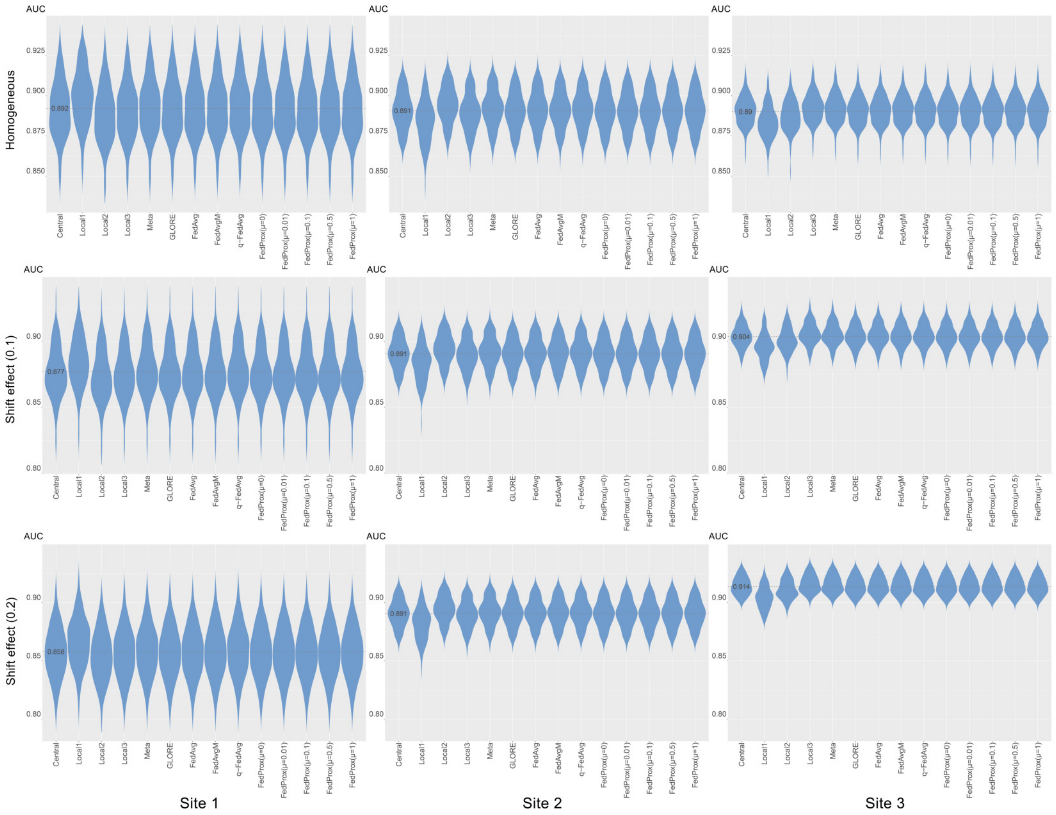

Figure S6: FL Model comparisons by prediction performance under shifting of effect size with relatively large sample size.

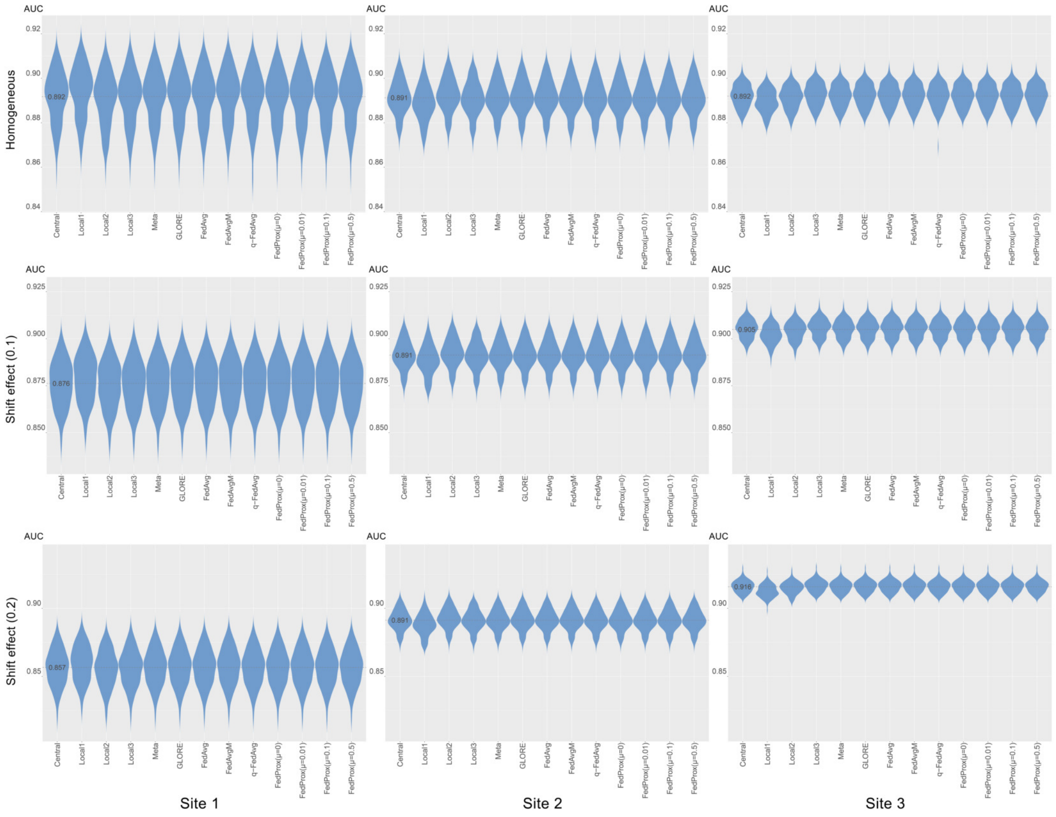

I.1.2 High Dimension

Figure S7: FL Model comparisons by prediction performance under shifting of covariate mean.

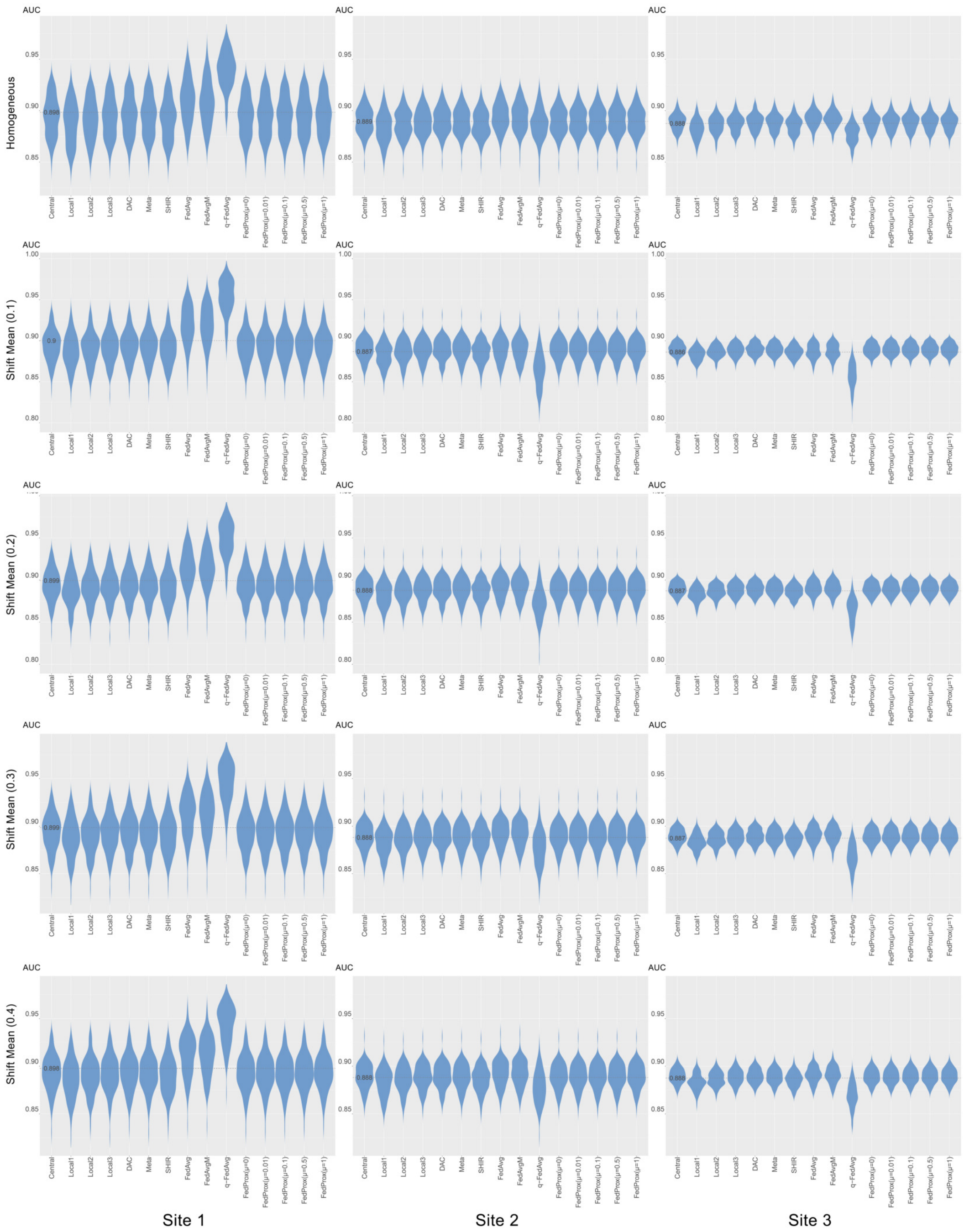

Figure S8: FL Model comparisons by prediction performance under shifting of SD.

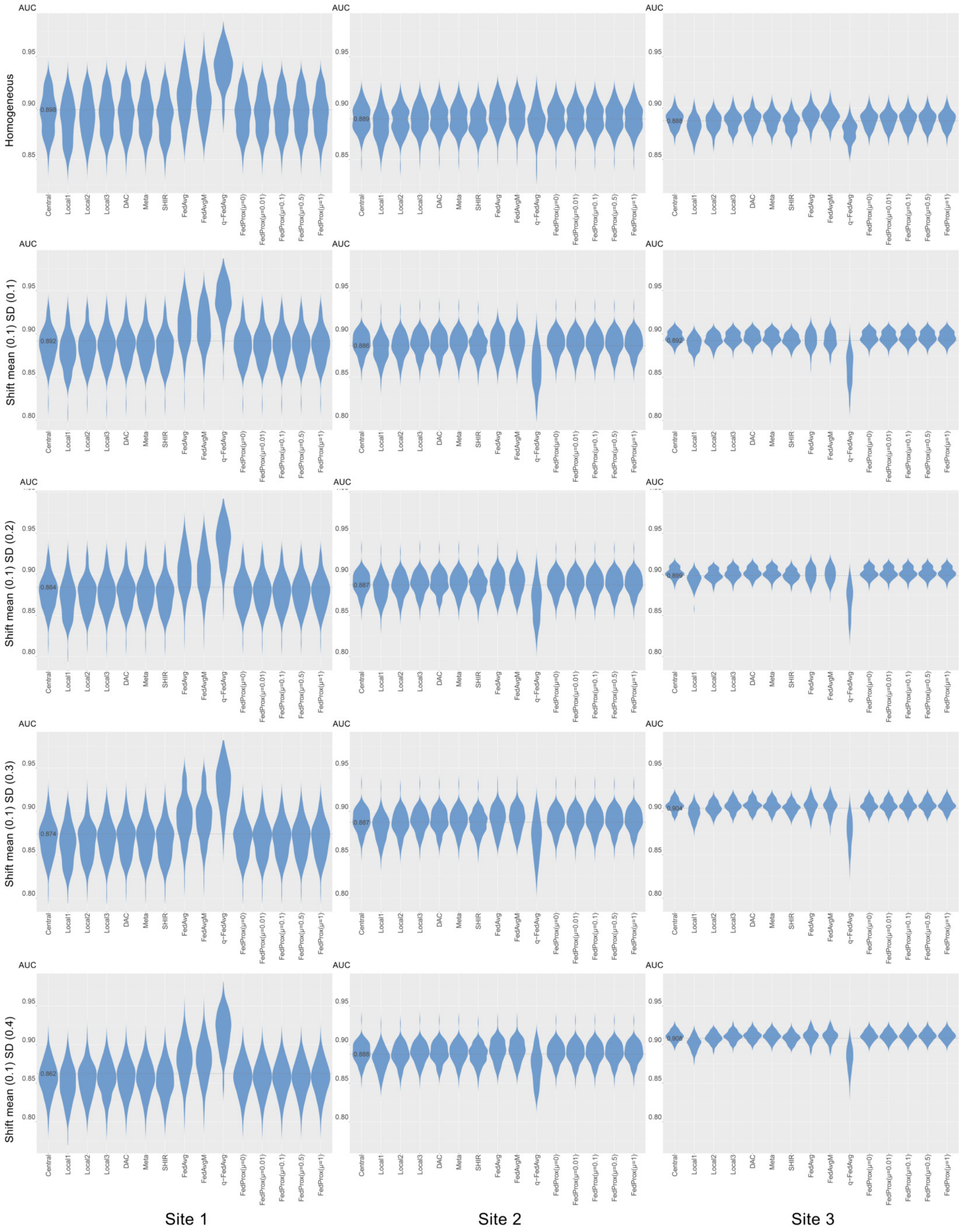

Figure S9: FL Model comparisons by prediction performance under shifting of effect size.

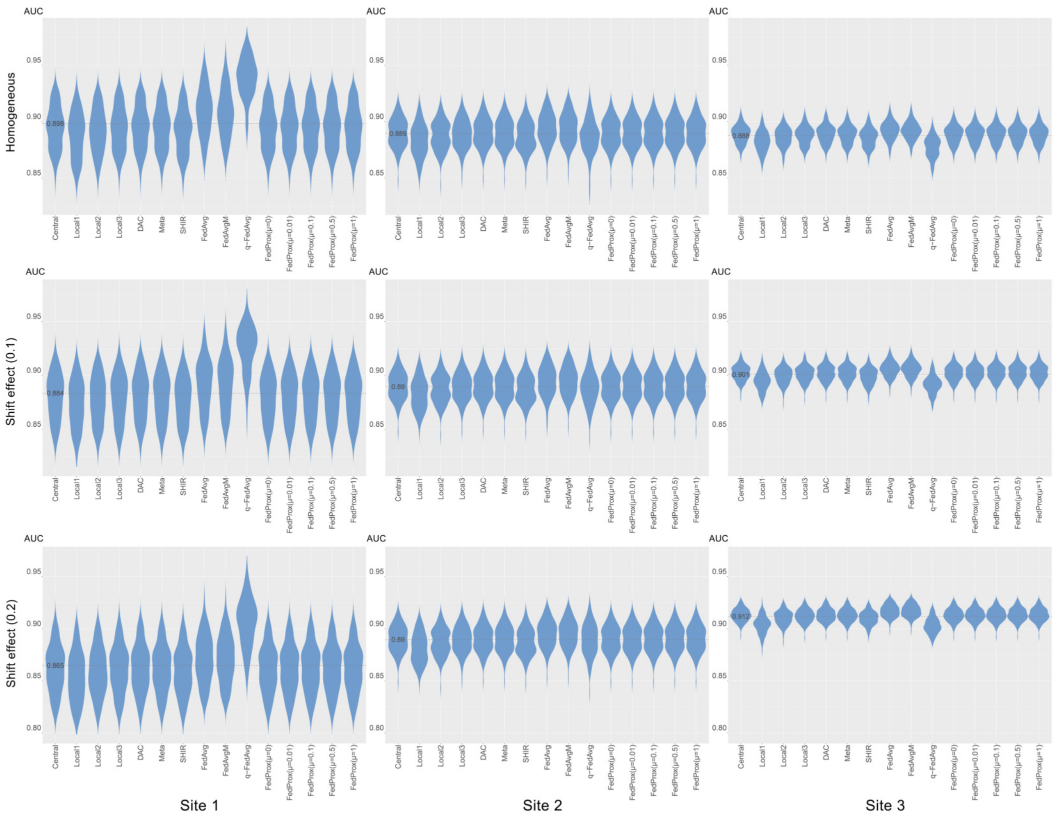

I.2 Point estimates

I.2.1 Low Dimension

Figure S10: FL Model comparisons by estimated coefficients under shifting of mean with relatively small sample size.

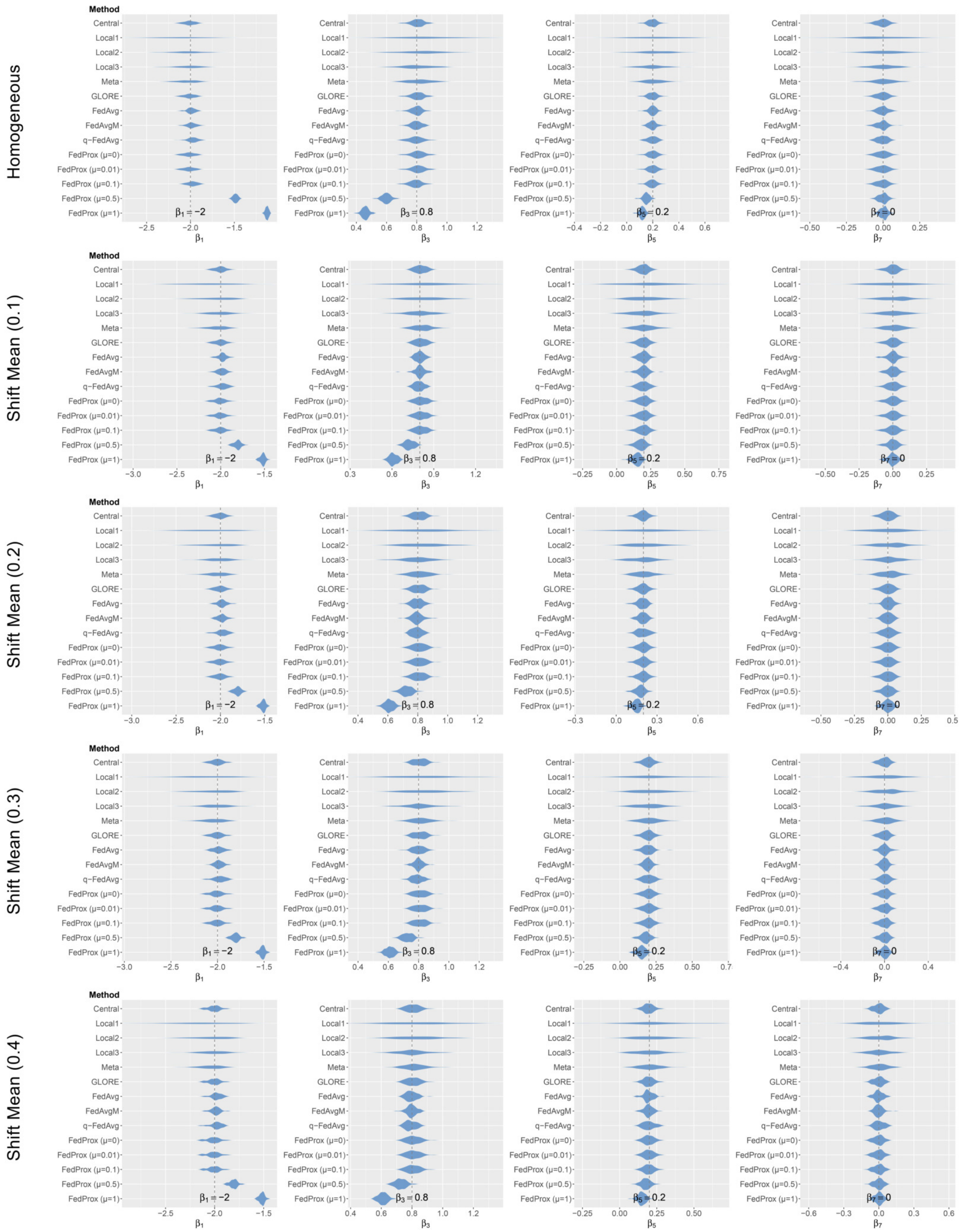

Figure S11: FL Model comparisons by estimated coefficients under shifting of mean with relatively large sample size.

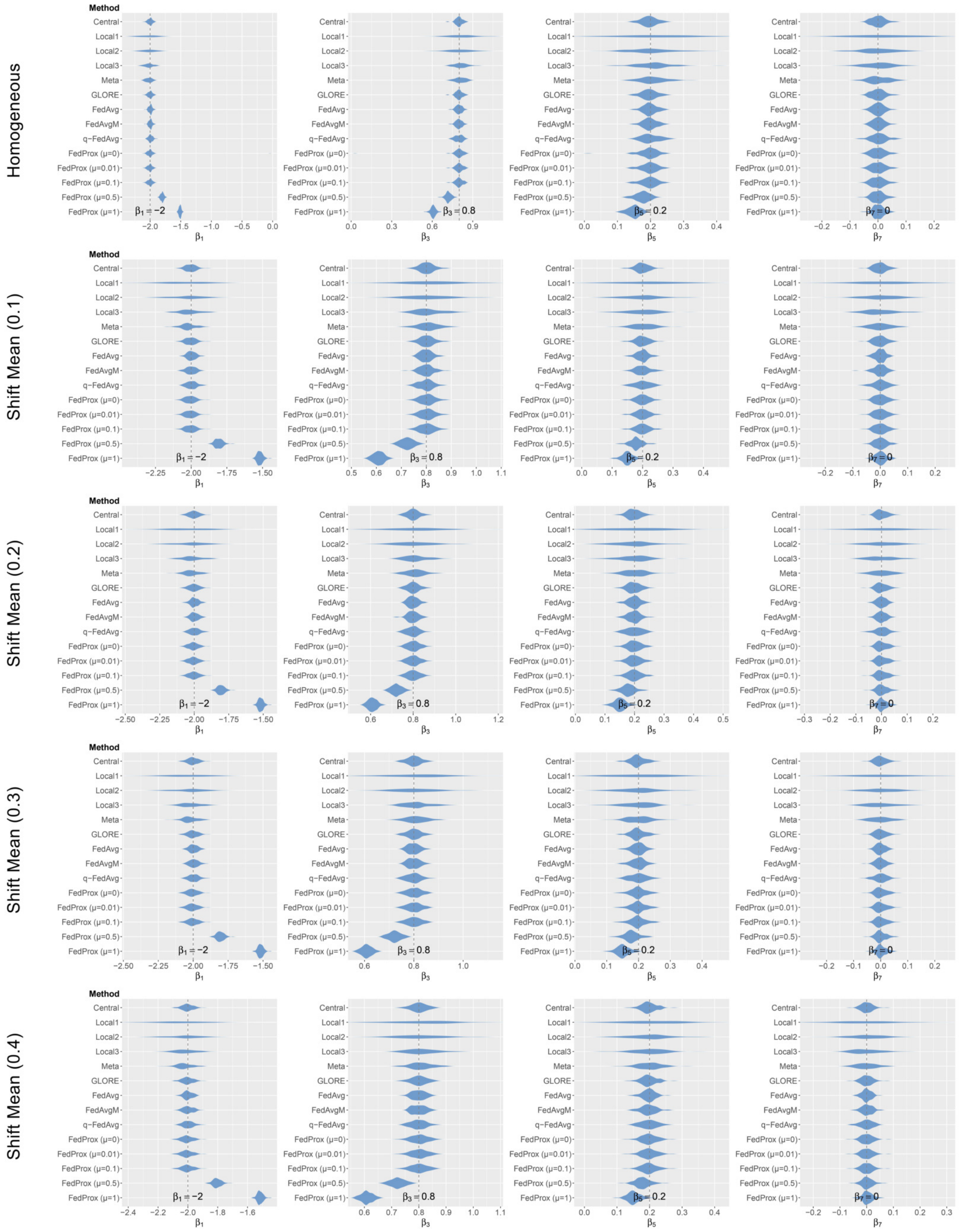

Figure S12: FL Model comparisons by estimated coefficients under shifting of SD with relatively small sample size.

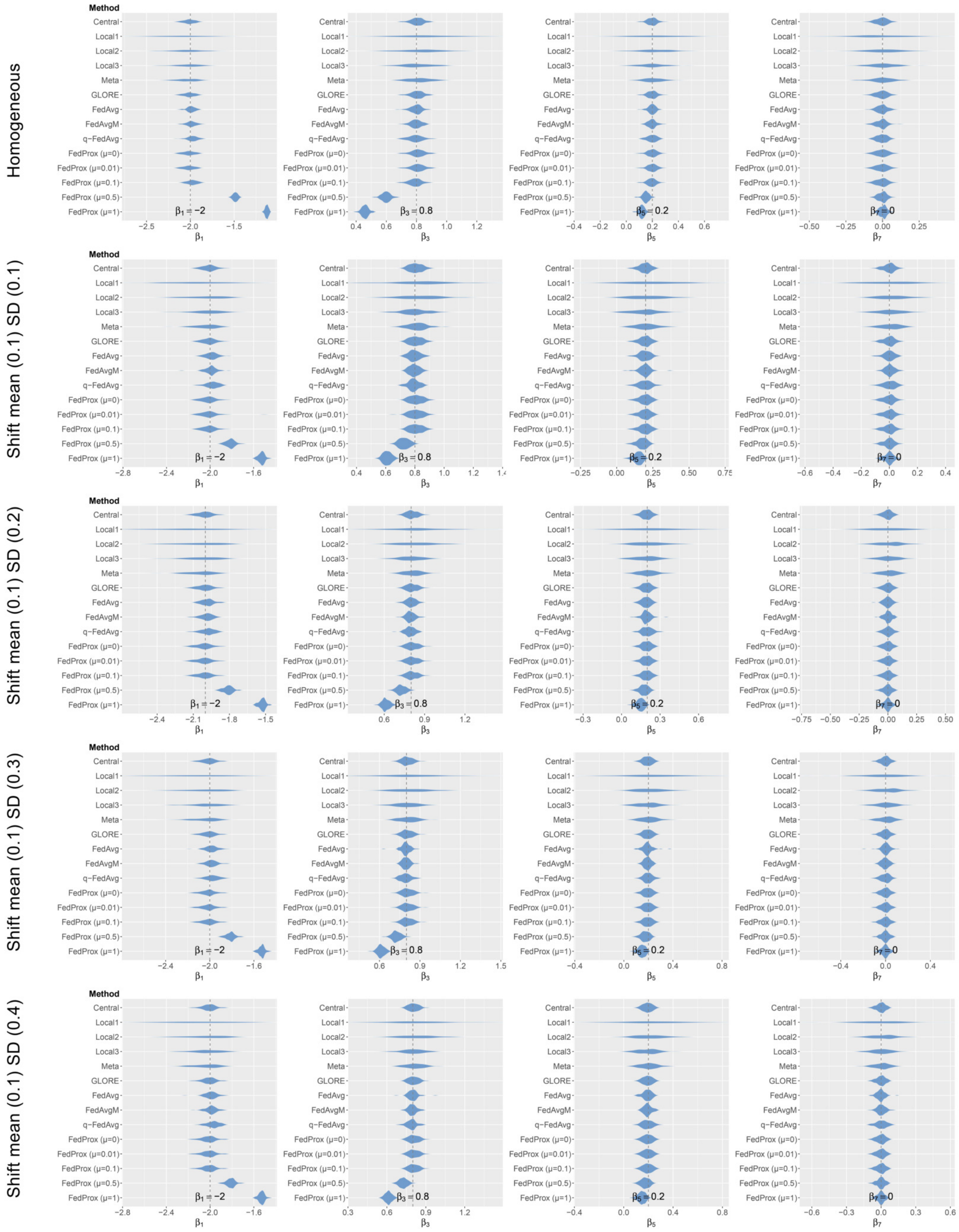

Figure S13: FL Model comparisons by estimated coefficients under shifting of SD with relatively large sample size.

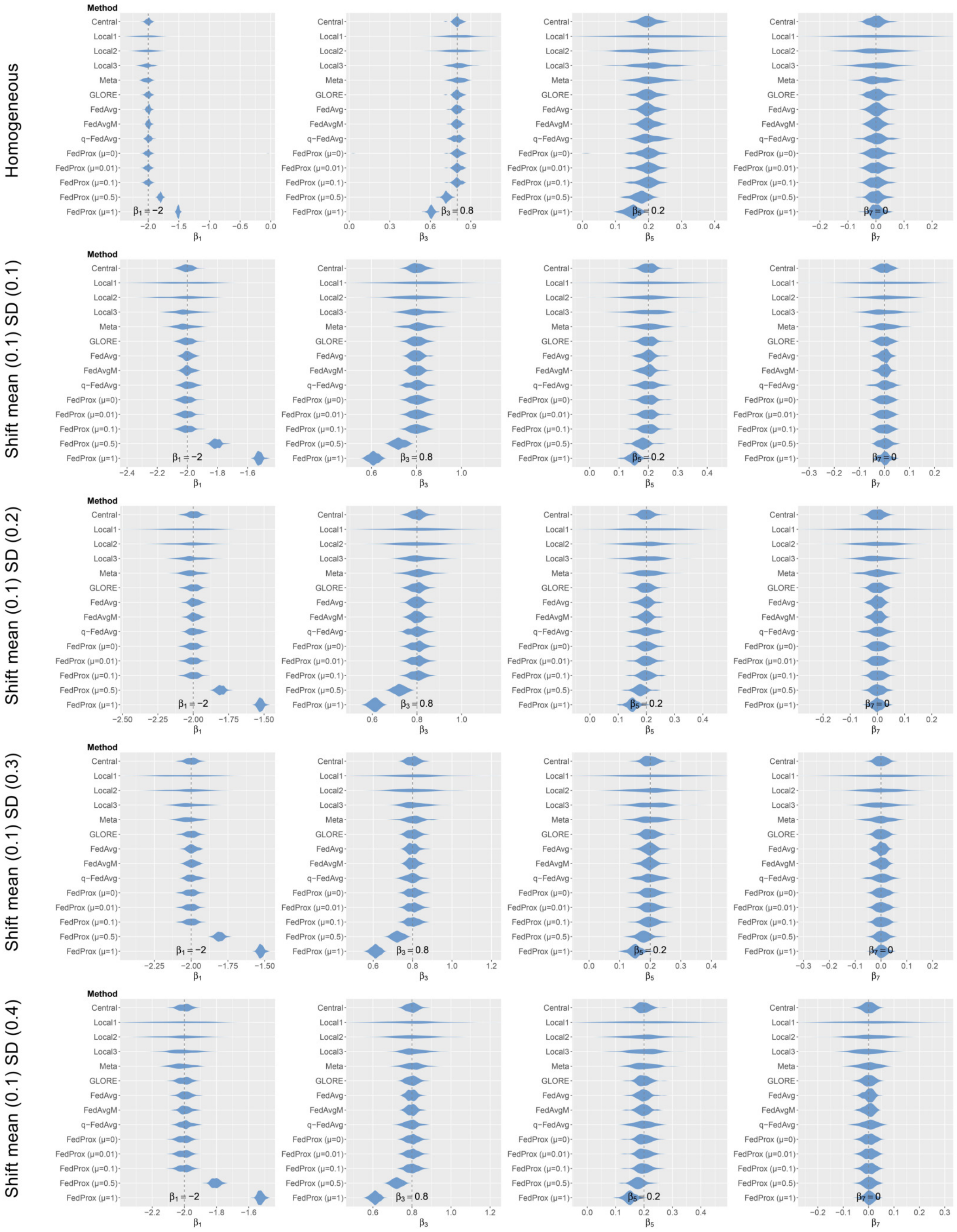

Figure S14: FL Model comparisons by estimated coefficients under shifting of effect size with relatively small sample size.

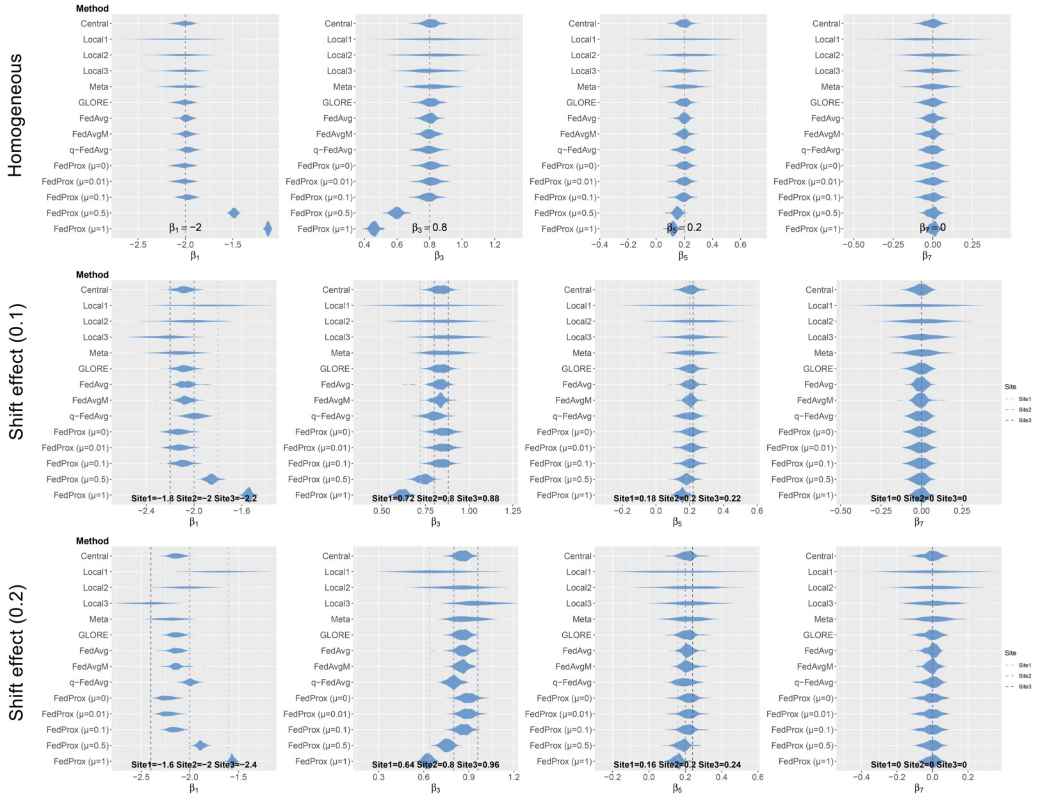

Figure S15: FL Model comparisons by estimated coefficients under shifting of effect size with relatively large sample size.

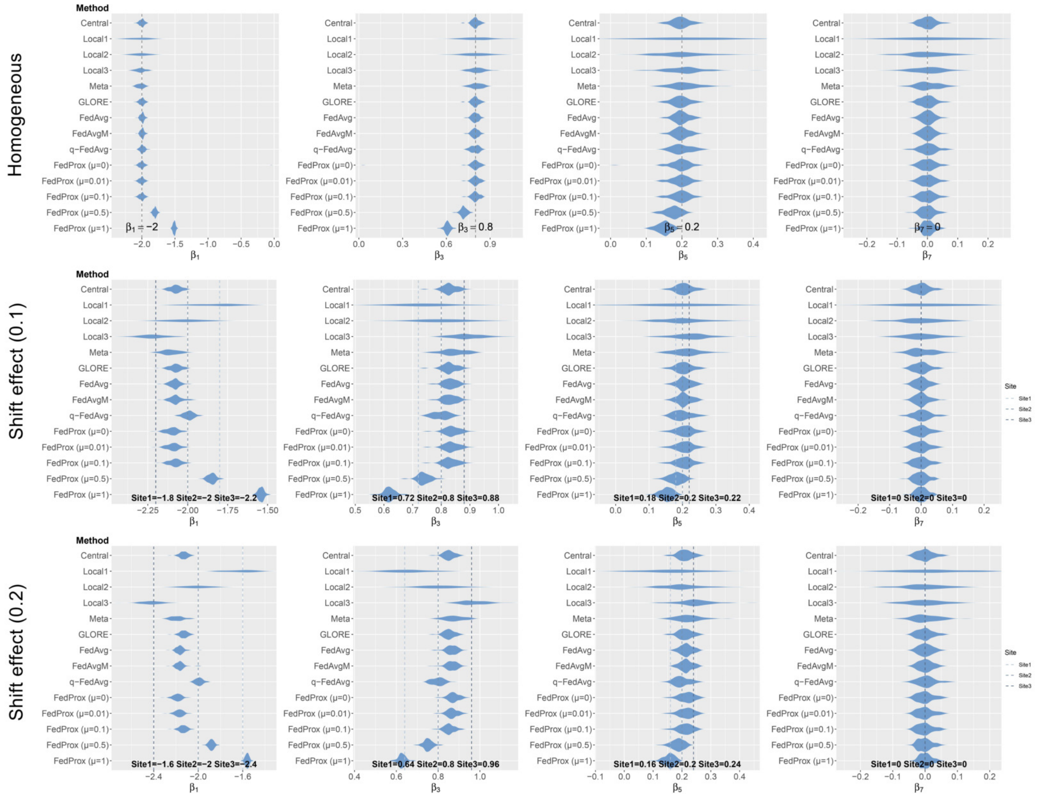

References

[1] Y. Wu, X. Jiang, J. Kim, and L. Ohno-Machado, "Grid binary logistic regression (glore): building shared models without sharing data," *Journal of the American Medical Informatics Association*, vol. 19, no. 5, pp. 758–764, 2012.

[2] T. Minka, "A comparison of numerical optimizers for logistic regression," March 2003. [Online]. Available: https://www.microsoft.com/en-us/research/publication/comparison-numerical-optimizers-logistic-regression/

[3] T. Cai, M. Liu, and Y. Xia, "Individual data protected integrative regression analysis of high-dimensional heterogeneous data," *Journal of the American Statistical Association*, vol. 117, no. 540, pp. 2105–2119, 2022.

[4] C. Hong, Y. Wang, and T. Cai, "A divide-and-conquer method for sparse risk prediction and evaluation," *Biostatistics*, vol. 23, no. 2, pp. 397–411, 2022.

[5] B. McMahan, E. Moore, D. Ramage, S. Hampson, and B. A. y. Arcas, "Communication-Efficient Learning of Deep Networks from Decentralized Data," in *Proceedings of the 20th International Conference on Artificial Intelligence and Statistics*, ser. Proceedings of Machine Learning Research, A. Singh and J. Zhu, Eds., vol. 54. PMLR, 20–22 Apr 2017, pp. 1273–1282. [Online]. Available: https://proceedings.mlr.press/v54/mcmahan17a.html

[6] T.-M. H. Hsu, H. Qi, and M. Brown, "Measuring the effects of non-identical data distribution for federated visual classification," *arXiv preprint arXiv:1909.06335*, 2019.

[7] T. Salazar, M. Fernandes, H. Araujo, and P. H. Abreu, "Fair-fate: Fair federated learning with momentum," *arXiv preprint arXiv:2209.13678*, 2022.

[8] T. Li, M. Sanjabi, A. Beirami, and V. Smith, "Fair resource allocation in federated learning," *arXiv preprint arXiv:1905.10497*, 2019.

[9] J. Bernstein, J. Zhao, K. Azizzadenesheli, and A. Anandkumar, "signsgd with majority vote is communication efficient and fault tolerant," *arXiv preprint arXiv:1810.05291*, 2018.

[10] T. Li, A. K. Sahu, M. Zaheer, M. Sanjabi, A. Talwalkar, and V. Smith, "Federated optimization in heterogeneous networks," *Proceedings of Machine learning and systems*, vol. 2, pp. 429–450, 2020.
